# Functionalizable Stereocontrolled Cyclopolyethers by Ring‐Closing Metathesis as Natural Polymer Mimics

**DOI:** 10.1002/anie.201805113

**Published:** 2018-06-20

**Authors:** Mohammed Alkattan, Joëlle Prunet, Michael P. Shaver

**Affiliations:** ^1^ EaStCHEM School of Chemistry University of Edinburgh Joseph Black Building David Brewster Road Edinburgh EH9 3FJ UK; ^2^ WestCHEM School of Chemistry University of Glasgow Joseph Black Building University Avenue Glasgow G12 8QQ UK

**Keywords:** cyclopolymers, polyethylene glycol, post-polymerization modification, ring-closing metathesis, stereocontrolled synthesis

## Abstract

Whereas complex stereoregular cyclic architectures are commonplace in biomacromolecules, they remain rare in synthetic polymer chemistry, thus limiting the potential to develop synthetic mimics or advanced materials for biomedical applications. Herein we disclose the formation of a stereocontrolled 1,4‐linked six‐membered cyclopolyether prepared by ring‐closing metathesis (RCM). Ru‐mediated RCM, with careful control of the catalyst, concentration, and temperature, selectively affords the six‐membered‐ring cyclopolymer. Under optimized reaction conditions, no metathetical degradation, macrocycle formation, or cross‐linking was observed. Post‐polymerization modification by dihydroxylation afforded a novel polymer family encompassing a poly(ethylene glycol) backbone and sugar‐like functionalities (“PEGose”). This strategy also paves the way for using RCM as an efficient method to synthesize other stereocontrolled cyclopolymers.

Control over the absolute configuration of a synthetic polymer main chain remains a significant challenge,^1^, ^2^ especially in light of the importance of this regularity in natural polymers.^3^ This stereoregularity of the main chain plays a significant role in shaping the three‐dimensional structure, and in‐turn influencing the biological function of the natural macromolecules. In addition, rings are embedded in the backbones of many natural polymers, which restricts the bond rotation around the stereogenic centers.^4^ These local conformational restrictions result in a specific compact structure along the polymer backbone: that is, the six‐membered cyclic structures of cellulose and amylose backbones (Figure 1) form linear and helical structures, respectively. However, synthetic polymers that are made of similar 1,4‐linked six‐membered rings that would mimic these secondary structures present a unique challenge to synthetic chemists, especially if conventional polymerization techniques are employed.^5^

**Figure 1:**
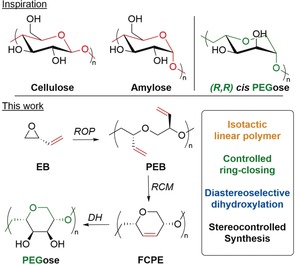
Biopolymers serving as an inspiration for stereocontrolled polymer synthesis, and a strategy to target the desired *cis*‐PEGose cyclopolymer.

Ring‐closing metathesis (RCM) has been predominantly used to prepare small cyclic molecules, including phosphine‐boranes, sulfides, amines, phenols, and oxazolines.^6^ The use of RCM in polymer synthesis, however, remains rare,^7^ with only a few examples of the preparation of polymeric nanoparticles,^8^ cyclic polymers,^9^ and cyclopolymers.^10^ We hypothesized that this under‐utilized post‐polymerization technique could be employed for the synthesis of cyclopolyethers to mimic the topology of polysaccharides, where the configuration of all the stereogenic centers present in the polymers is controlled (Figure 1). While the broad functional group tolerance of metathesis catalysts suggests a broad reaction scope, poly(ethylene glycol) backbones are especially interesting given their role as gold standard stealth polymers in drug delivery.^11^ We thus envisaged a sequence of ring‐opening polymerization (ROP), ring‐closing metathesis (RCM), and dihydroxylation (DH), as shown in Figure 1. An initial ROP of 3,4‐epoxy‐1‐butene (EB) would afford polyepoxybutene (PEB), with the chirality of the parent epoxide leading to stereogenic control in the linear polymer. Ring‐closing metathesis would then give a 1,4‐linked functionalizable cyclopolyether (FCPE). Further functionalization, specifically diatereoselective dihydroxylation (DH), would produce a new stereocontrolled polymer that we have called “PEGose”, as it has the structural features of both sugars and PEG, with the glycosidic bond of amylose replaced by a strong ether link.

For this structural control, it is imperative to start with enantiopure EB. Atactic PEB would lead to a non‐stereocontrolled cyclopolymer, where the 1,4‐links would be indiscriminately *cis* (amylose‐like) or *trans* (cellulose‐like; Figure 2). Furthermore, the subsequent dihydroxylation reaction would not be diastereoselective on the *trans* 1,4‐disubstituted six‐membered rings. On the other hand, dihydroxylation of the *cis*‐cyclopolymer will occur exclusively from the top face, which is not hindered by the two substituents.

**Figure 2:**
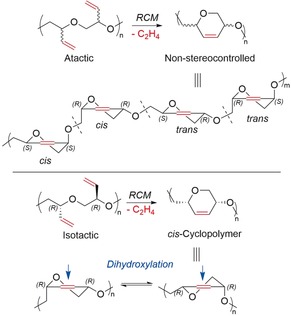
Influence of polymer tacticity on RCM and resultant polymer stereochemistry in *cis*‐ and atactic‐FCPE.

Ring‐opening and ring‐closing reactions were first explored with the commercially available racemic 3,4‐epoxy‐1‐butene monomer. Although PEB has been prepared by a number of routes from the EB monomer,^12^, ^13^ our optimized reaction conditions used tetraphenylporphyrin aluminum chloride [(TPP)AlCl] as an initiator^14^ for the bulk polymerization of EB at ambient temperature [Eq. 1, see Table S1 in the Supporting Information].
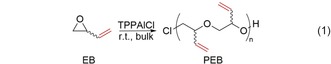



The ^13^C NMR spectrum of the resultant PEB showed two signals for the stereogenic carbon atom, thus confirming the expected atacticity of the produced PEB (*a*‐PEB), as the catalyst is not stereoselective. Controlled low‐molecular‐weight polymers were produced, with *M*_*n*,GPC_ values of 2100 and 3200, respectively, and *Ð*<1.2. Although RCM on *a*‐PEB will not give a stereocontrolled cyclopolyether, it was used to establish the optimum RCM conditions. Although little difference in molecular weight was observed by gel‐permeation chromatography (GPC) for the lowest molecular weight PEB samples, because of overlap with eluent peaks, the higher molecular weight samples (*M*_*n*,GPC_ 3200) showed a clear loss of molecular weight on ring closing, correlating well with the loss of a single ethylene molecule per repeat unit (Table 1). Optimal ring‐closing conditions for PEB included the use of 5 mol % of the second‐generation Hoveyda–Grubbs (HG2) catalyst at high concentrations of the polymer (≥0.2 m with respect to the monomer unit) in 1,2‐dichloroethane (1,2‐DCE; Table 1). Note that poor conversions were achieved with the first‐generation Grubbs catalyst, likely because of the lower reactivity and thermal stability (see Table S2).^15^

**Table 1:** RCM of *a*‐PEB. 
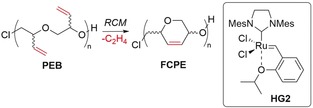

Entry^[a]^	[Olefin]	*M*_*n*,st_^[b]^	*Ð*_st_^[b]^	Conv. [%]^[c]^	*M*_*n*,th_^[d]^	*M_n_* ^[b]^	*Ð* ^[b]^
1	0.2	3200	1.19	99	2540	2500	1.21
2	0.4	3200	1.19	99	2540	2900	1.3

[a] Reactions performed in 1,2‐DCE at reflux with 5 mol % of the HG2 catalyst for 72 h. [b] *M_n_* and *Ð* determined by GPC versus uncorrected PS standards. [c] Determined by ^1^H NMR spectroscopy by integration of the olefin signals of the produced polymer. [d] *M*_*n*,th_=PEB *M*_*n*,GPC_ × 0.8 because of the expected loss of one C_2_H_4_ per monomer unit.

When concentrations were kept at 0.2 m (Table 1, entry 1), no cross‐linking was observed, and the polymer dispersity and viscosity remained similar. However, at higher concentrations (0.4 m), competing ring‐closing and polymer cross‐linking occurs, as evidenced by the formation of a high‐molecular‐weight shoulder in the GPC traces (see Figure S1).^16^ This new polymer represents the first synthetic cyclopolyether prepared, but its inherent atacticity prevents any overall topological control. Thus, isotactic‐rich PEB (*i*‐PEB) was synthesized by ROP of the *R* enantiomer of EB (95:5 e.r.), which was prepared from racemic EB by Jacobsen's hydrolytic kinetic resolution (see page 3 in the Supporting Information).^17^ The behavior of the isotactic polymer towards RCM was directly compared to that of its atactic derivative under the previously optimized conditions (Table 2).

**Table 2:** RCM of PEB with HG2 catalyst.

Entry^[a]^	PEB *M_n_* ^[b]^	*Ð* ^[b]^	*T*_g_ [°C]^[c]^	*t* [days]	FCPE		*Ð* ^[b]^	*T*_g_ [°C]^[c]^
					*M*_*n*,th_^[d]^	*M_n_* ^[b]^		
*rac*	4380	1.18	−56	5	3500	2700	1.22	−26
*R*	3940	1.15	−54	7	3150	2600	1.19	−11

[a] [Olefin]=0.2 m; the reaction conversion in 1,2‐DCE at reflux with 5 mol % HG2 catalyst was monitored daily until >99 % by ^1^H NMR spectroscopy. [b] *M_n_* and *Ð* determined by GPC versus uncorrected PS standards. [c] Determined by differential scanning calorimetry. [d] *M*_*n*,th_=PEB *M*_*n*,GPC_ × 0.8.

The kinetics of the cyclization of the enantiomerically pure monomer were significantly slower than for the racemic monomer. Plotting the reaction kinetics (Figure 3 and Figure S2) showed that the cyclization reaction progressed quickly in the beginning, with 94 % of the pendent vinyl groups forming cross‐links within 30 min for both the atactic and isotactic derivatives. The metathesis reaction then significantly slowed down, especially for the more conformationally rigid isotactic derivative. This profile suggests a mechanism originally proposed by Coates and Grubbs:^10^ 1) a fast stage when the catalyst randomly closes adjacent olefins until only isolated olefins remain, and 2) a slow stage when the rings rearrange along the chain until all the olefins are cyclized. This requires that the cyclized olefins can undergo further metathetic reactions, thus enabling a reopening and exchange of the product rings.^18^ The two‐stage reactivity is showcased through the RCM optimization, with the first stage completed in a similarly short time regardless of the solvent used (1,2‐DCE, DCM, THF, CHCl_3_) or catalyst loading (2–5 %; see Tables S3–S6).

**Figure 3:**
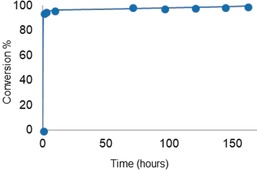
The RCM of *i*‐PEB, *M_n_*,_GPC_ 3940 and *Đ* 1.15, kinetic profile at 0.2 m using 5 % HG2 catalyst in 1,2 DCE under reflux monitored by ^1^H NMR spectroscopy.

An illustration of this mechanism can be observed in the ^13^C NMR spectra of both the atactic and isotactic FCPE and PEB (Figure 4), which demonstrate that greater than 99 % of the olefins of PEB are cyclized (Figure 4 B,E). In the atactic FCPE (Figure 4 B), the new olefin peaks appear as broad, overlapping resonances (*δ*=125–132 ppm), which reflects the different ring configurations along the polymer backbone. On the other hand, *i*‐FCPE showed only two sharp olefin resonances (Figure 4 E), thereby confirming the stereocontrolled structure of the polymer (Figure 2, *cis*‐cyclopolymer). However, the spectrum of *i*‐FCPE after 94 % conversion (Figure 4 D), which was taken after 30 min, showed the 6 % of uncyclized isolated olefin signals (*δ*=118.3 and 135.6 ppm) and several cyclic olefin signals (*δ*=125–132 ppm).

**Figure 4:**
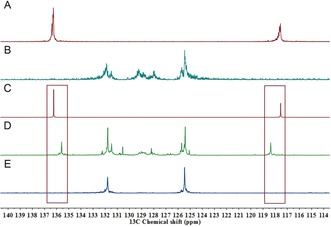
^13^C NMR spectra of the olefin region in CDCl_3_ of: A) *a*‐PEB, B) *a*‐FCPE, C) *i*‐PEB, D) metathesis product of *i*‐PEB after 30 min (94 % conversion), and E) metathesis product of *i*‐PEB after 7 days (>99 % conversion).

Most of these cyclic olefin peaks were not observed at the end of the reaction (after 7 days), which purports that in the initial metathesis stage when the catalyst randomly closes the olefins, different ring sizes were formed (Figure 5). In the subsequent slow stage, the rings rearrange along the chain until only the most thermodynamically stable six‐membered rings are present.

**Figure 5:**
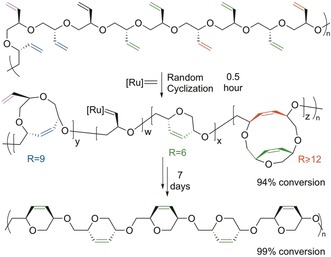
Proposed kinetic mechanism of the RCM of *i*‐PEB.

Fixing the free rotation of the pendent olefins through RCM has an impact on the glass transition temperature (*T*_g_) in both *a*‐ and *i*‐PEB. The organized structure of *i*‐FCPE has a significantly higher *T*_g_ value than *a*‐FCPE (−11 °C from −26 °C). This is consistent with the presence of cycles hindering segmental chain mobility in both structures.

To prepare the PEGose polymer, *i*‐FCPE was dihydroxylated under mild conditions using *N*‐methylmorpholine *N*‐oxide (NMO) and OsO_4_ as a catalyst. This second post‐polymerization functionalization was diastereoselective, as OsO_4_ attacks on the less hindered side of the ring (Figure 2), as demonstrated by ^13^C NMR spectroscopy (Figure 6). The dihydroxylation produces a unique stereocontrolled polymer structure, with a hydrophilic surface (*cis*‐diols) opposite the hydrophobic backbone. This distinctive structure could have potential applications in biomaterials, blood storage, or drug delivery, with face polarity shaping the surface chemistry and self‐assembly.^19^, ^20^ While amylose, (C_6_H_10_O_5_)_*n*_, and PEGose, (C_6_H_10_O_4_)_*n*_ have similar monomer units, PEGose is connected with an additional methylene bridge, which gives more flexibility to the polymer backbone. Circular dichroism (CD) was used to determine the influence of this CH_2_ unit on the secondary structure of the PEGose. Indeed, PEGose and amylose have the same prominent negative bands at *λ*=182 nm (Figure S25), which shows that this new PEGose has an extended pseudohelical structure similar to amylose.^21^ Efforts to gain complementary X‐ray characterization of this self‐assembly are ongoing.

**Figure 6:**
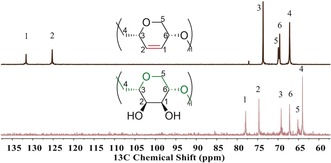
^13^C NMR spectra of *i*‐FCPE (top) and *cis*‐PEGose (bottom) in CDCl_3_ and D_2_O, respectively.

Although excess NMO affords complete dihydroxylation of the double bonds, the reaction also offers the ability to adjust the polymer polarity by limiting this co‐oxidizing reagent. Reducing the NMO loading from 1.1 to 0.8 equivalents dramatically alters the polarity and solubility of the resultant polymer (Table 3), thereby offering a secondary tuning for biomedical applications and leaving sites remaining for further functionalization or drug conjugation.^22^ Whereas the parent polymer is soluble in organic solvents and the fully dihydroxylated polymer is freely soluble in water and DMSO, this strategy allows for a broad range of polymer polarities to be accessed.

**Table 3:** Controlled dihydroxylation to form *cis*‐PEGose. 
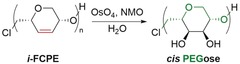

Entry	[Olefin]/[NMO]	Con. [%]^[a]^	Soluble in	Insoluble in
1	1:1.1	>99	H_2_O, DMSO	DMF
2	1:0.9	91	DMF, MeOH	acetone
3	1:0.8	80	acetone	EtOAc, CH_2_Cl_2_

[a] Determined by ^1^H NMR spectroscopy through integration of the olefin signals relative to the polymer signals.

In conclusion, we have shown that RCM of linear, stereoregular polymers with pendent olefins can be used to prepare cyclopolymers with excellent control over the ring size. Further functionalization of the latent olefin groups by dihydroxylation provides sugar‐like structures with a poly(ethylene glycol) backbone that leads to a new PEGose architecture. The isotactic linear PEB leads, after RCM, to a cyclic polymer with well‐defined *cis* substitution patterns. By taking advantage of the diastereoselectivity of the subsequent dihydroxylation reaction, we were able to create a cyclopolymer where the configuration of all the stereogenic centers is controlled, and which mimics the natural amylose. This new platform offers significant potential for future functionalization, drug conjugation, and biomedical mimicry, and is a significant focus of our future work, as is expanding this idea to other polymer backbones.

## Conflict of interest

The authors declare no conflict of interest.

## Supporting information

As a service to our authors and readers, this journal provides supporting information supplied by the authors. Such materials are peer reviewed and may be re‐organized for online delivery, but are not copy‐edited or typeset. Technical support issues arising from supporting information (other than missing files) should be addressed to the authors.

Supplementary
