# Supplementary material for: Functionalizable Stereocontrolled Cyclopolyethers by Ring‐Closing Metathesis as Natural Polymer Mimics
Source: Angew Chem Int Ed Engl. 2018 Jun 20;57(39):12835–9. doi: 10.1002/anie.201805113 (PMC6175094; doi:10.1002/anie.201805113)
Supplement: Supplementary file 1 — Supplementary [file ANIE-57-12835-s001.pdf]

Angewandte Chemie
Eine Zeitschrift der Gesellschaft Deutscher Chemiker

Supporting Information

Functionalizable Stereocontrolled Cyclopolyethers by Ring-Closing Metathesis as Natural Polymer Mimics

Mohammed Alkattan, Joëlle Prunet, and Michael P. Shaver**

anie_201805113_sm_miscellaneous_information.pdf

Table of Contents

General Considerations 2

Materials 2

Instrumentation 2

Experimental Procedures and Characterization Data 3

Nuclear Magnetic Resonance (NMR) Spectra 12

IR Spectroscopy 20

Differential scanning calorimetry 21

Inductively coupled plasma 22

Circular dichroism (CD) spectra 23

References 24

General Considerations

Experiments involving air- and moisture-sensitive compounds were performed in oven-dried glassware either under a nitrogen atmosphere using an MBraun LABmaster sp glovebox system equipped with a −35 °C freezer and [H_2O] and [O_2] analyzers or using standard Schlenk techniques. Dichloromethane (DCM) and hexane were obtained from an Innovative Technologies solvent purification system incorporating columns of alumina and copper catalysts. 1,2 Dichloroethane (DCE) and chloroform were purified by distillation under nitrogen and dried via Å3 molecular sieves. Tetrahydrofuran (THF) was exposed for further purification procedures by sodium metal and benzophenone at 70°C overnight, then distilled under on inert atmosphere and all these solvents were degassed by three freeze−pump−thaw cycles prior to use.

Materials

Grubbs first-generation catalyst, Hoveyda−Grubbs second-generation catalyst, 3,4-epoxy-1-butene, 1,2-epoxy-5-hexene, osmium tetraoxide, *N*-methylmorpholine-*N*-oxide, trioctylamine, Sephadex LH-20 and G-25 were purchased from Sigma-Aldrich. (*R,R*)-(−)-*N,N'*-*bis*(3,5-di-*tert*-butylsalicylidene)-1,2-cyclohexanediaminocobalt (II) was purchased from Acros Organics. 3,4-epoxy-1-butene and 1,2-epoxy-5-hexene were dried over calcium hydride by refluxing for 18 h, and distillation under a nitrogen atmosphere was used prior to being degassed by three freeze−pump−thaw cycles.

Instrumentation

Gel permeation chromatography (GPC) was performed using a Malvern Instruments Viscotek 270 GPC Max triple detection system with 2× mixed bed styrene/DVB columns (300 × 7.5 mm) in THF at 35 °C at a flow rate of 1 mL.min^{-1} and an injection volume of 100 µL. Samples for analysis were pre dissolved in THF at a concentration of ~1−2 mg/mL. ^{1}H-NMR spectra were recorded at 298 K using BrukerAsance spectrometers (500 MHz). ^{13}C- and Dept- 135 NMR spectra were recorded using BrukerAsance spectrometers (126 MHz). 2D NMR analyses (COSY and HSQC) were recorded using BrukerAsance spectrometers (500 MHz). $CDCl_3$ and D_2O were used as solvent for NMR analyses. Differential scanning calorimetry (DSC) was carried out using a TA Instruments DSC Q2500 instrument, through a heat/ cool/heat cycle between −90 and 100 °C at a rate of 10 °C min^{-1} Values of T_g, was obtained from the second heating scan. The enantiomeric excess (ee) was determined by chiral HPLC Agilent 1200. IR spectroscopy was taken by Shimadzu FTIR-8400S. The level of metals residues in the polymers were detected by Perkin Elmer Optima 5300 DV Inductively Coupled Plasma - Optical Emission Spectrometry (ICP-OES). The circular dichroism (CD) spectra were measured using a Jasco J-810 spectropolarimeter.

Experimental Procedures and Characterization Data

Synthesis of Tetraphenylporphyrin aluminum chloride (TPP)AlCl [1]

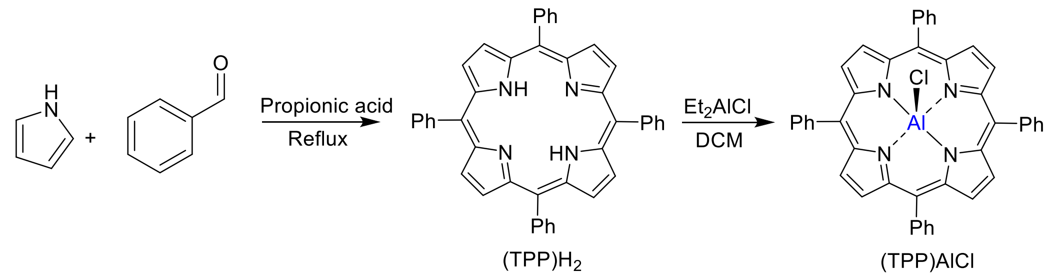

5,10,15,20-Tetraphenylporphine (TPP)H_2 was synthesized from freshly distilled pyrrole (4.56 g, 68 mmole) and benzaldehyde (7.21 g, 68 mmol) in propionic acid (300 mL) under reflux for 4 h, and the crude product precipitated upon standing overnight at room temperature, was filtered and then washed by water and methanol. The obtained crystals were recrystallized from $CHCl_3$/MeOH (1/2 in v/v) and dried overnight under vacuum to give $TPPH_2$ (23%) yield as purple crystals.

^{1}H-NMR (500.2 MHz, *$CDCl_3$*) δ = 8.84 (s, 8H), 8.22 (d, 8H), 7.76 (s, 12H), -2.76 (s, 2H).

In glovebox, (TPP)H_2 (1.00 g, 1.63 mmol) was dissolved in DCM (30 mL) and 1 M diethylaluminium chloride solution in hexane (1.31g, 1.82 mmol) was added slowly. After 3 h, the volatiles were removed, and the product was washed by hexane and dried overnight to give (TPP)AlCl (96 % yield) as bright purple crystals.

^{1}H-NMR (500.2 MHz, *$CDCl_3$*) δ = 9.09 (s, 8H), 8.20 (s, 8H), 7.83–7.70 (m, 12H).

Synthesis of R isomer of 3,4 epoxy-1-butene [2]

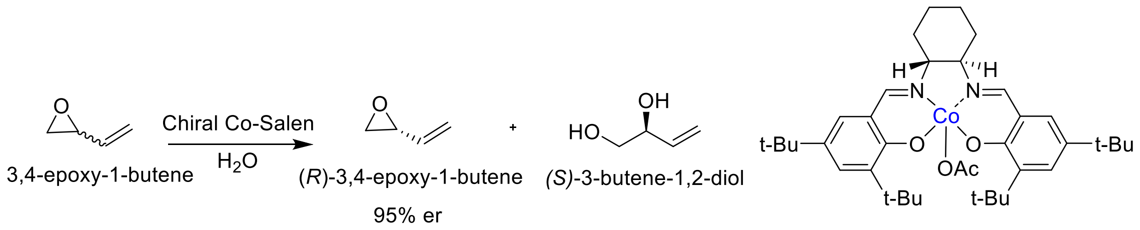

The catalyst's ligand (*R,R*)-*N,N'-bis*(3,5-di-*tert*-butylsalicylidene)-1,2-cyclohexanediaminocobalt(II), (1.00 g, 1.65 mmol, 0.015 equiv) in 8 mL of toluene was treated with AcOH (1 mL) and stirred in air for 3 h. The crude mixture was left under full vacuum for 3 h. The catalyst residue obtained was dissolved in racemic 3,4-epoxy-1-butene (7.74 g, 110 mmol). The solution was cooled to 0 °C and H_2O (1.4 mL, 78 mmol, 0.71 equiv) was added dropwise. After 96 h at r.t., (*R*)-3,4-epoxy-1-butene (2.51 g, 35.7 mmol, 32%) was isolated by vacuum transfer into a cooled catch flask. The enantiomeric excess (ee) was determined by chiral HPLC analysis of the 2-napthylsulfide derivative (obtained by ring opening with 2-napthalenethiol in MeOH using 1 equiv of triethylamine (TEA) at 0 °C and direct analysis of the product obtained, Chiralcel® OD, 95:5 hexanes:*i*-PrOH, 1 mL / min, 230 nm, t_R(minor) = 13.27 min, t_R(major) = 14.51 min).

Synthesis of poly(epoxy butene) (PEB) via Ring Opening Polymerization (ROP)

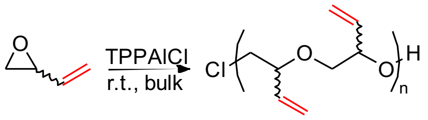

In glove-box, cold 3,4-epoxy-1-butene (racemic or *R*-isomer) (140 mg 2.0 mmol) were added to TPPAlCl (0.02 mmol 13.5 mg) and stirred at the ambient temperature for a selected time. DCM (1 ml) was then added and the mixture quenched by 5 mL of 1 M HCl/MeOH and stirred for a further 1 h. The volatiles were removed under reduced pressure and the residue dissolved in MeOH:DCM 1:1 (1 mL), and the resulting suspension was filtered to remove insoluble initiator residues. The polymer solution was then purified by size exclusion chromatography using Sephadex LH-20 and MeOH:DCM 1:1 as an eluent. The volatiles were removed and the polymer was dried under vacuum overnight to give a brown oil of atactic PEB and a brown gel of isotactic PEB.

Table S1: Ring-opening polymerization of racemic 3,4-epoxy-1-butene (EB).

Entry	$[EB]_0:[A]_0:[B]_0$ [a]	Time days	Con.(%) [b]	$M_{n,th}$ [c]	M_n [d]	Đ [d]
1	36:1:0	2	>99	2560	3200	1.19
2	50:1:0	3	>99	3540	4400	1.18
3	70:1:0	5	96	4750	5400	1.17
4	100:1:0	7	90	6345	6550	1.19
5*	170:1:1	2	82	9670	4650	1.27

[a] [A]: TPPAlCl initiator, [B]: MAlBP; [b] Determined by ^{1}H-NMR spectroscopy of polymer/monomer peaks integration; [c] M_n theoretical = M_w(monomer)× (%Con.) + M_w(HCl); [d] M_n and Đ determined by GPC vs uncorrected PS standard.

^{1}H-NMR (500.2 MHz, *$CDCl_3$*) δ = 5.77-5.69 (m, br, 1H), 5.33-5.13 (m, br, 2H), 3.91-4.02 (m, br, 1H), 3.60-3.38 (m, br, 2H).

^{13}C-NMR (125.8 MHz, *$CDCl_3$*) δ = 136.2, 117.8, 80.8 and 72.1.

*****Due to the long reaction time, a Lewis acid such as methylaluminum *bis* (2,4,6-tri-*tert*-butylphenolate) [MAlBP] was introduced as a common accelerator.[3] However, we noticed from the GPC data and ^{1}H-NMR spectrum that a side reaction occurred between MAlBP and (TPP)AlCl led to new initiators.[4] For this reason, the molecular weight in this case (entry 5) was almost half the one expected and the MAlBP residues became the polymer's end groups. This was not noticed when (TPP)AlCl was used alone.

Synthesis of Functionalizable Cyclopolyethers (FCBE) via Ring-Closing Metathesis (RCM)

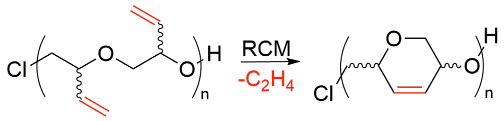

All reactions were conducted in evacuated ampules of volumes at least four times greater than the solution volume. In 20 mL ampule, atactic PEB or isotactic PEB (60 mg, 0.86 mmol based on the monomer molecular weight) were stirred for 15 minutes at 84 °C in 1,2-dicloroethane (DCE) (3.8 ml). Then, second-generation of Hoveyda-Grubbs catalyst (27 mg 0.043 mmol, 5 mol. %) in DCE (0.5 mL) was added slowly under nitrogen. After 30 min a static vacuum was applied carefully. The reaction progression was monitored by ^{1}H-NMR spectroscopy daily and after completion the reaction mixture was cooled to room temperature and 100 equivalents of DMSO (305 µL, 4.3 mmol) was added and the resulting mixture stirred for 1 h. The volatiles were then removed and the residue was dissolved in MeOH:DCM 3:1 (1 mL). The polymer was purified by size exclusion chromatography using Sephadex LH-20 and MeOH:DCM 3:1 as an eluent. The volatiles were removed and then the polymer was dried at 60 °C under vacuum overnight to give a brown rubber.

Atactic:

^{1}H-NMR (500.2 MHz, *$CDCl_3$*) δ = 6.16–5.64 (m, br, 2H, -C**H**=C**H**-), 4.37–3.99 (m, br, 2H, -C**H**O-), 3.92–3.30 (m, br, 4H, -C**H_2**-).
^{13}C-NMR (125.8 MHz, *$CDCl_3$*) δ = 132.0–124.0 (br, -**C**=**C**-), 74.1–73.2 (br, -**C**HO-), 71.2–70.4 (br, -**C**H_2-), 70.4–69.3 (br, -**C**HO-), 68–66.6 (br, -**C**H_2-).

Isotactic:

^{1}H-NMR (500.2 MHz, *$CDCl_3$*) δ = 6.32–5.71 (m, br, 2H, -C**H**=C**H**-), 4.37–3.99 (m, br, 2H, -C**H**O-), 3.92–3.30 (m, br, 4H, -C**H_2**-).
^{13}C-NMR (125.8 MHz, *$CDCl_3$*) δ = 131.8 (-**C**=C-), 125.5 (-C=**C**-), 73.9 (-**C**HO-), 70.1 (-**C**H_2-), 69.8 (-**C**HO-), 67.4 (-**C**H_2-).

1- Catalyst type:

Table S2: RCM of atactic PEB using first-generation Grubbs and second-generation Hoveyda−Grubbs catalysts.

[Olefin][a] M	Con.(%)[b] 1st Grubbs	Con.(%)[b] 2nd H-G
0.05	43	80
0.1	61	90
0.15	68	93

[a] PEB $M_{n,GPC}$ 2100 and Đ 1.19 in DCM under reflux with [Olefin]:[Catalyst] = 20:1; Time 43 hours; [b] Determined by ^{1}H-NMR spectroscopy of olefin peaks integration of the produced polymer.

2- Polymer Concentration:

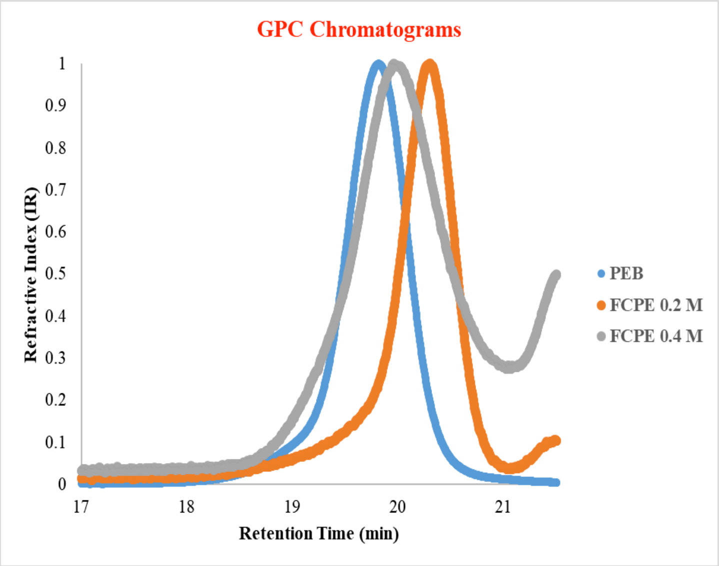

Figure S1: Gel Permeation Chromatography (GPC) chromatograms of PEB and FCPE at 0.2 and 0.4 M concentrations.

3- Solvent and temperature:

To demonstrate the solvent and temperature effect, the reaction was studied also at different temperatures using solvents that easily dissolve PEB and are compatible with the used metathesis catalyst. The results demonstrated that using relatively high boiling point solvent is required to accelerate the reversed metathesis stage, so 1,2 dichloroethane (DCE) showed the fastest conversion rate comparing with DCM, THF and chloroform (Table S3). However, since the results in table S3 did not show a significant conversion difference in the initial fast cyclization stage, the reaction was repeated at different times in the slow reversed metathesis stage using DCM and DCE solvents and the atactic and isotactic PEB (Table S4).

Table S3: RCM of *a*-PEB in differ-ent solvents at different temperaturs.[a]

Solvent	Temp. (°C)	Con.[b] (%)
DCM	40	92
$CHCl_3$	62	90
THF	62	92
DCE	84	94

[a] [PEB] = 0.2 M, $M_{n,GPC}$ 3200 and Đ 1.19; Time 30 minutes; [Olefin]:[2nd H-G] = 20:1; [b] Determined by ^{1}H-NMR spectroscopy of olefin peaks integration of the produced polymer.

Table S4: RCM of *a*-PEB and *i*-PEB at two differ-ent temperaturs.[a]

Polymer	Solvent	Temp. (°C)	Time (days)	Con.[b] (%)
Atactic	DCM	40	5	98
Isotactic	DCM	40	7	95
Isotactic	DCM	40	14	97
Atactic	DCE	84	5	99
Isotactic	DCE	84	7	99

[a][PEB] = 0.2 M; *a*-PEB-$M_{n,GPC}$ 4400 and Đ 1.18; *i*-PEB-$M_{n,GPC}$ 3940 and Đ 1.15; [Olefin]:[2nd H-G] = 20:1; [b] Determined by ^{1}H-NMR spectroscopy of olefin peaks integration of the produced polymer.

4- Catalyst ratio:

Running the reaction at two different catalysts ratio (5 mol. % and 2 mol. %) illustrated that the reaction rate is highly dependent on the catalyst loading.

Table S5: RCM of atactic PEB at two different catalyst ratio of 2nd H−G catalyst.

Time (hours)	Cat. Ratio (%)	
	5	2
T_1 (0.5)	94	92
T_2 (22)	97	95
T_3 (43)	98.5	96
T_4 (72)	>99	96

[a] [PEB] = 0.2 M, $M_{n,GPC}$ 3200 and Đ 1.19 in DCE under reflux; [b] Determined by ^{1}H-NMR spectroscopy of olefin peaks integration of the produced polymer.

5- RCM of different molecular weight polymers:

Table S6: RCM of different molecular weight of atactic PEB.[a]

a-PEB M_n[b]	Đ [b]	Time (days)	Con.(%)[c]	*a*-FCPE $M_{n,th}$[d]	*a*-FCPE M_n[b]	Đ [b]
3180	1.19	3	>99	2540	2500	1.21
4380	1.18	5	>99	3500	2700	1.22
5380	1.17	7	>99	4300	3100	1.22
6540	1.19	8	>99	5230	3700	1.26

[a] The reaction was carried out in DCE under reflux and [PEB] = 0.2 M; [Olefin]:[2nd H-G] = 20:1; [b] M_n and Đ determined by GPC vs uncorrected PS standard; [c] Determined by monitoring the reaction daily by ^{1}H-NMR spectroscopy; [d] *a*-FCPE $M_{n,}$ theoretical = *a*-PEB $M_{n,GPC}$ × 0.8 (due to 20% mass loss of ethylene per cyclomonomer unit) + [$M_w(CH_2)$ × unreacted olefin %].

6- The kinetic profile:

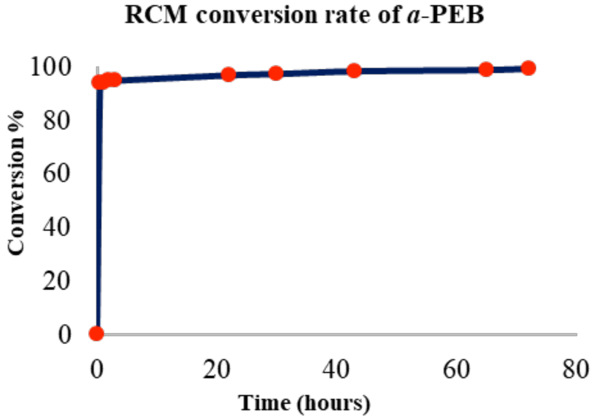

Figure S2: The RCM of atactic PEB, $M_{n,GPC}$ 3200 and Đ 1.19, kinetic profile at 0.2 M using 5% of 2nd H-G catalyst in 1,2 DCE under reflux monitored by ^{1}H-NMR spectroscopy.

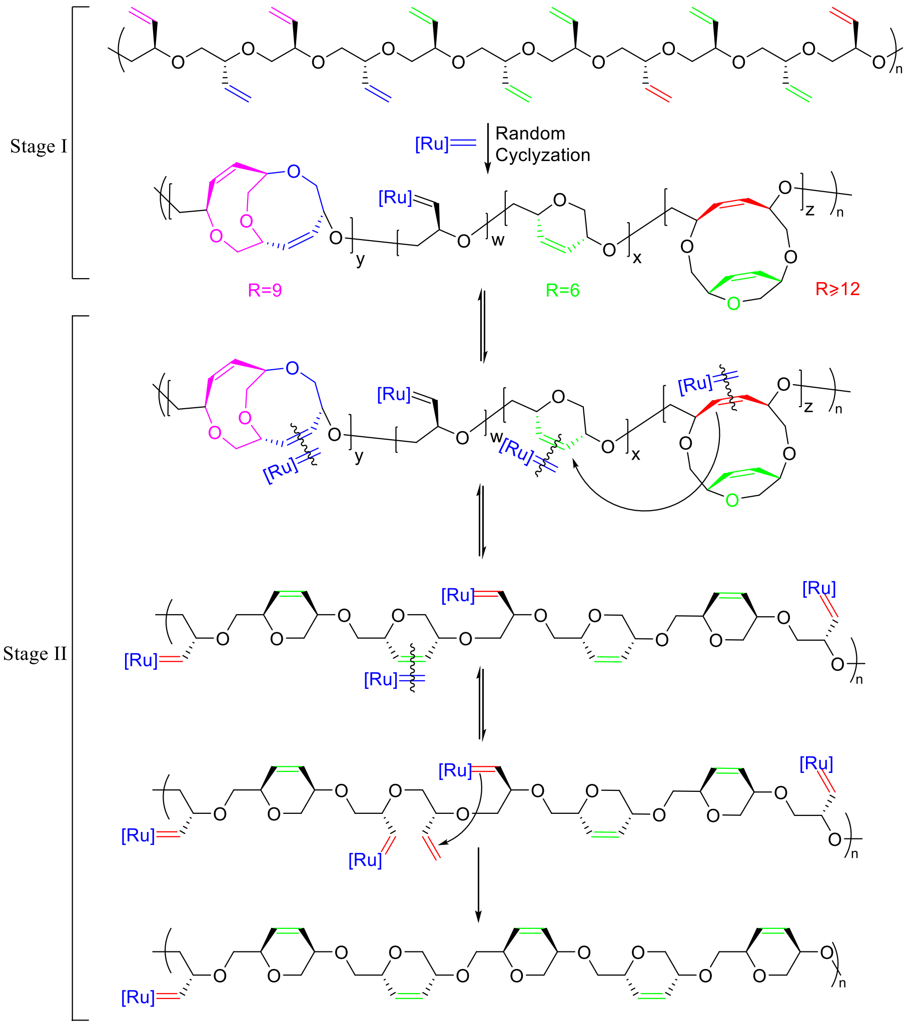

Figure S3: Proposed kinetic mechanism of RCM of poly (epoxybutene).

Olefin Cross vs. Ring-Closing Metathesis:

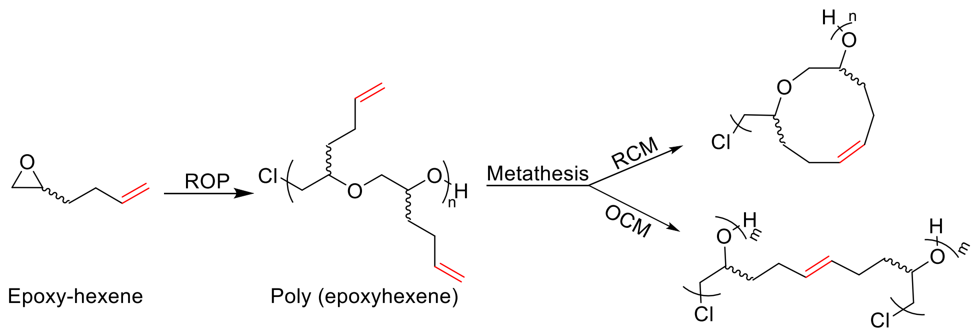

To demonstrate whether larger rings (9, 12, etc.) can be formed in the initial metathesis stage we performed the following comparison study on atactic poly (epoxybutene) (PEB) and atactic poly (epoxyhexene) (PEB) (Table S7). It was noticed in the first 30 min of the reaction that PEB tends to form rings rapidly, as exhibited by a decrease in the apparent molecular weight (M_n) with similar dispersity Đ. However, PEH showed a different behaviour; it demonstrated mainly cross-linking metathesis (intermolecular), as exhibited by an increase in the apparent molecular weight (M_n) and a broadening of the molecular weight distribution Đ (from 1.15 to ~ 3) (Table S7, figures S8 and S9). Consequently, RCM of a polymer with longer pendent olefins to form medium (8–11 membered rings) is more difficult. The medium rings not only have a kinetic disadvantage, but also suffer from unfavourable transannular interactions.[5]

Table S7: Comparison between PEB and PEH behaviours in metathesis reaction.[a]

Polymer	M_n [b]	Đ [b]	[C] M	Time (h.)	Con.[c] (%)	$M_{n,th}$ [d]	M_n [b]	Đ [b]
PEB	4380	1.18	0.2	0.5	92	3610	2750	1.22
PEH	4880	1.15	0.2	0.5	88	4340	14700	2.95
PEB	4380	1.18	0.015	12	90	3650	3700	1.21
PEH	4880	1.15	0.015	12	94	4270	14625	3.31

[a] The reaction was carried out in DCM under reflux; [Olefin]:[2nd H-G] = 20:1; [b] M_n and Đ determined by GPC vs uncorrected PS standard; [c] Determined by ^{1}H-NMR spectroscopy; [d] M_n theoretical = $M_{n,GPC}$ × 0.8 or 0.834 (due to 20% or 16.67% mass loss of ethylene per cyclomonomer unit of PEB and PEH, respectively) + [$M_w(CH_2)$ × unreacted olefin %].

Dihydroxylation of FCPE for PEGose

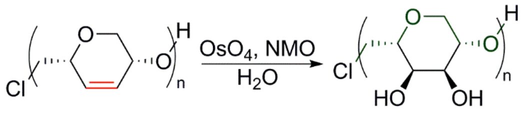

In a 50-mL round bottom flask, *i*-FCPE (112 mg, 1.00 mmol of the monomer unit) was dissolved in acetone:water (6 mL, 2:1). Then, 1.1 equivalent of dried *N*-methylmorpholine-*N*-oxide (129 mg, 1.10 mmol) was added to the mixture. The reaction mixture was stirred in an ice bath, and then ~100 μL of OsO_4 solution (1% in water) was added slowly and stirred further for 2 h. The flask was loosely capped, warmed up to the ambient temperature, and stirred overnight until the mixture became almost homogeneous. To quench the reaction, sodium sulfide (3 mL of saturated solution in methanol) was added and continued stirring for 1 h. The mixture was filtered to remove any insoluble residue and the volatiles were evaporated. The polymer solution was purified by SEC using Sephadex G-25 and H_2O as an eluent for fully dihydoxylated PEGose and H_2O:MeOH 3:1 for 90 and 80% conversion of PEGose. Then, the polymer was dried in a freeze-dryer to give a black colored gel. The polymer was further purified to reduce the Os residues level by extraction using trioctylamine (TOA) as a scavenger (Page S22).

***R,R cis*-PEGose**

^{1}**H-NMR** (500.2 MHz, *D_2O*) δ = 5.61–5.63 (m, br, 8H).

^{13}**C- NMR** (125.8 MHz, *D_2O*) δ = 77.9 (-H**C**OH-), 74.7 (-H**C**OH-), 69.2 (-**C**H_2-), 67.2 (-**C**HO-), 65.1 (-**C**HO-), 64.0 (-**C**H_2-).

Nuclear Magnetic Resonance (NMR) Spectra

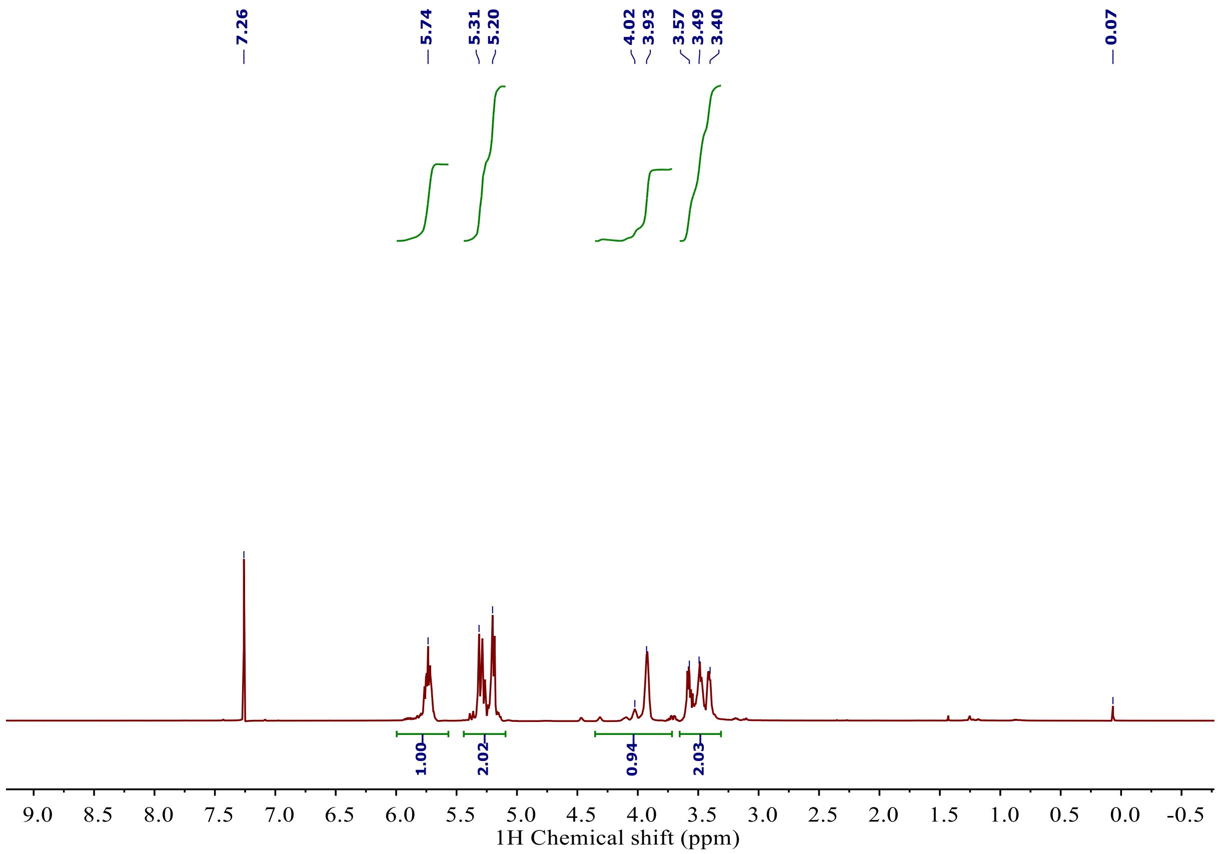

Figure S4: ^{1}H-NMR (*CDCl$_3$*, 500.2 MHz) of atactic poly (epoxybutene).

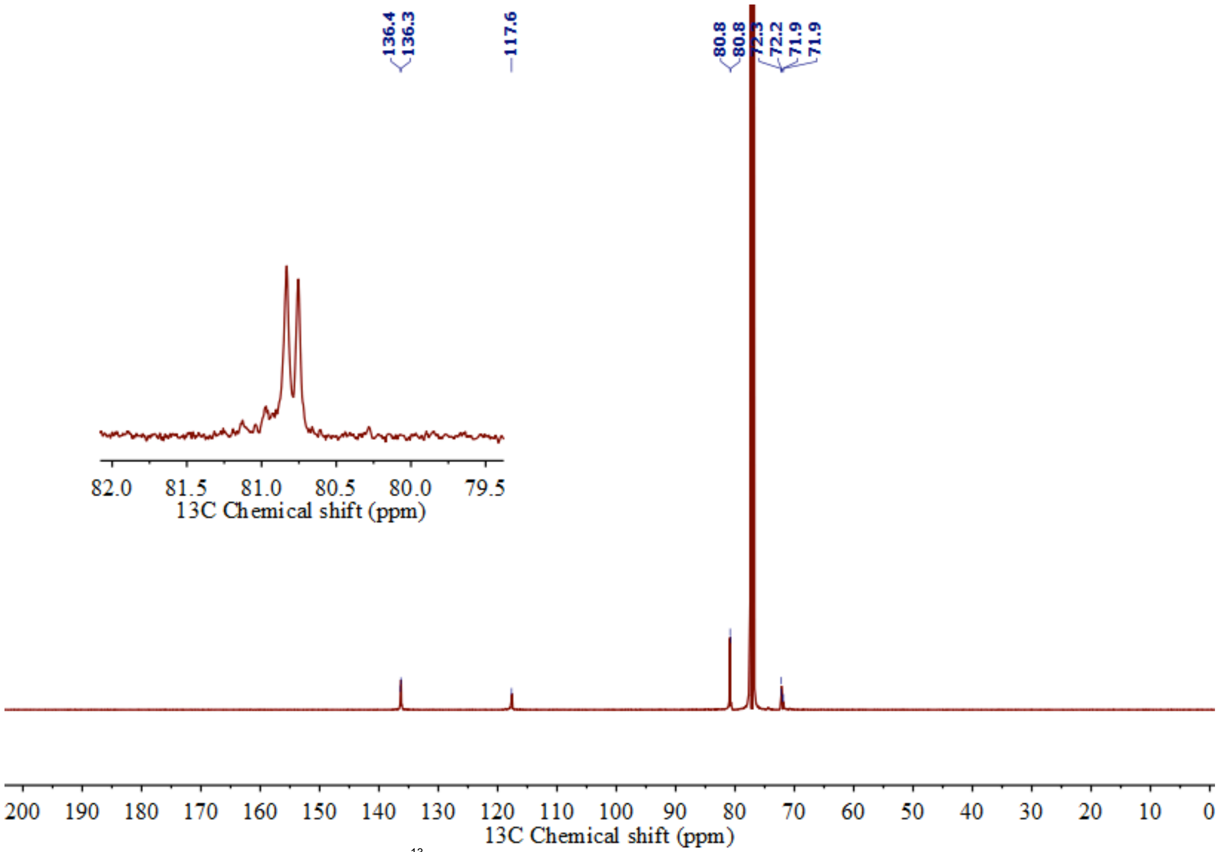

Figure S5: ^{13}C-NMR (*CDCl$_3$*, 125.8 MHz) of atactic poly (epoxybutene).

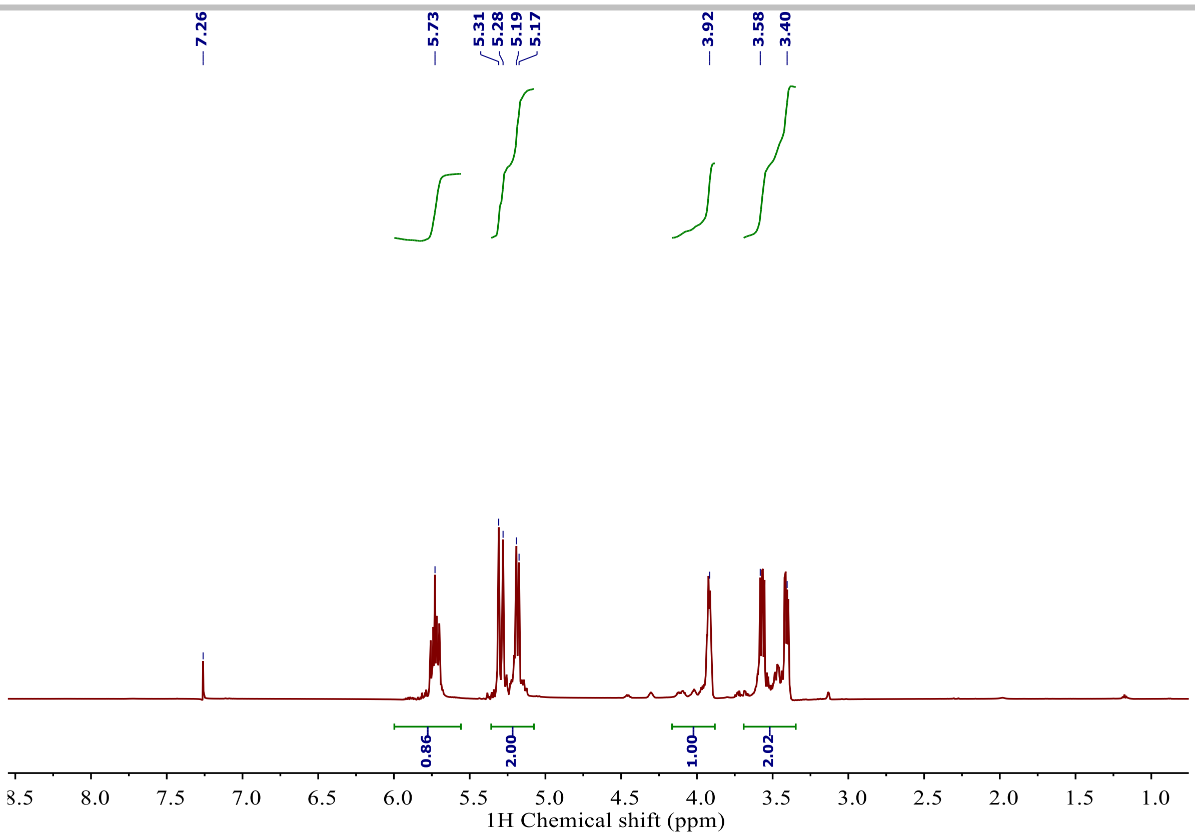

Figure S6: ^{1}H-NMR (*CDCl$_3$*, 500.2 MHz) of isotactic poly (epoxybutene).

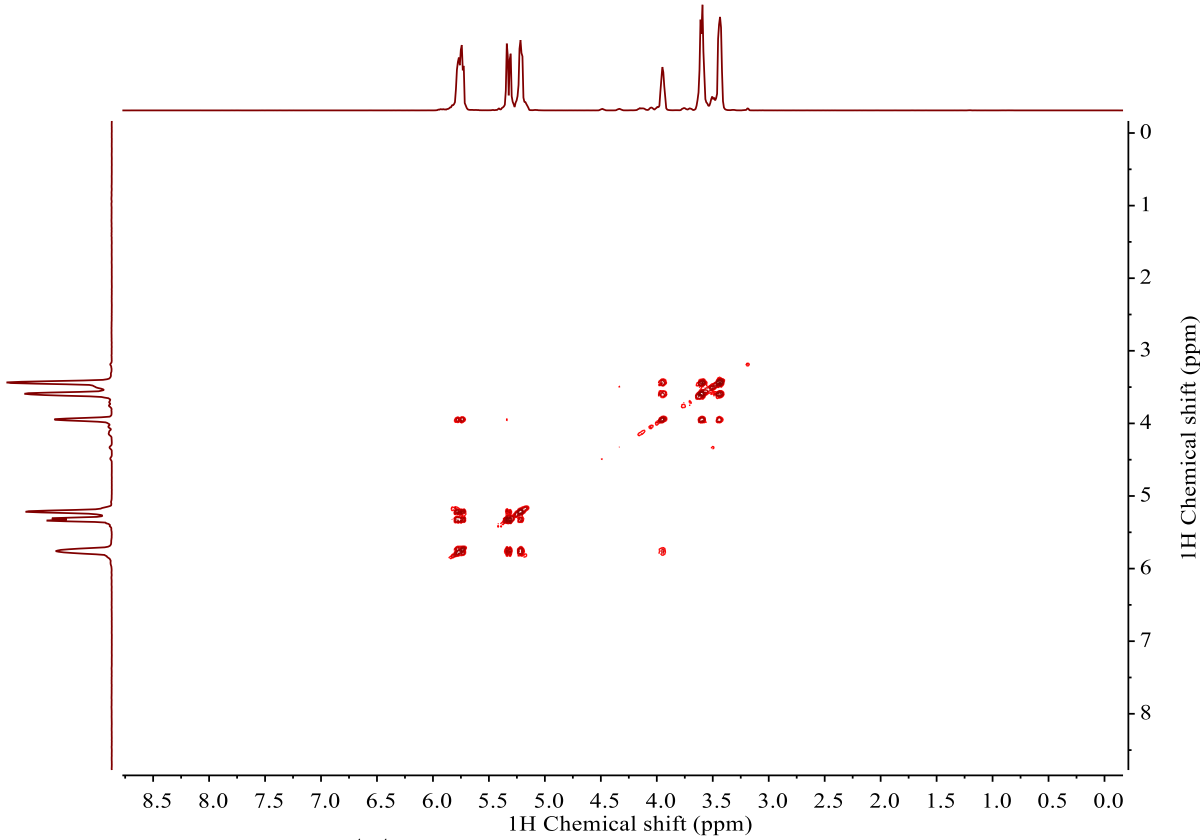

Figure S7: ^{1}H-^{1}H COSY NMR (*CDCl$_3$*, 500.2 MHz) of isotactic poly (epoxybutene).

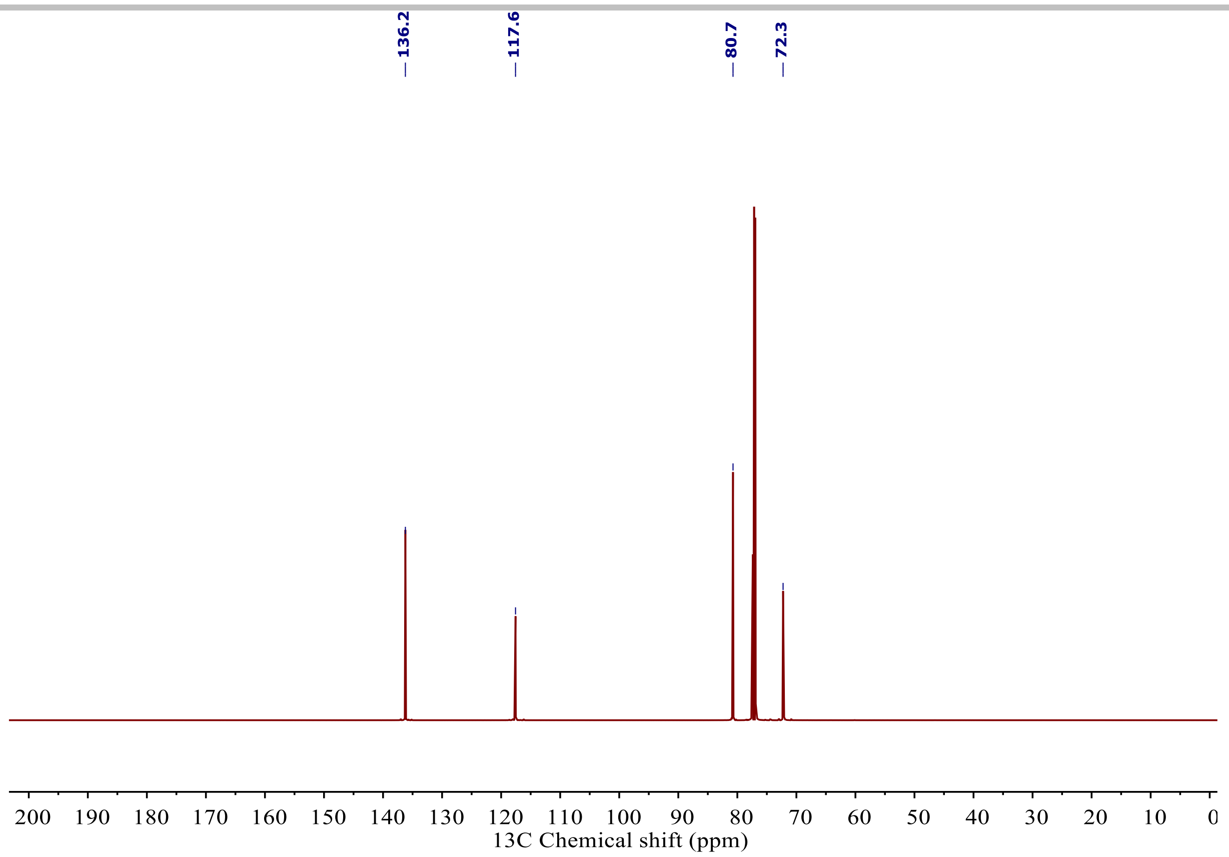

Figure S8: ^{13}C-NMR (*CDCl*$_3$, 125.8 MHz) of isotactic poly (epoxybutene).

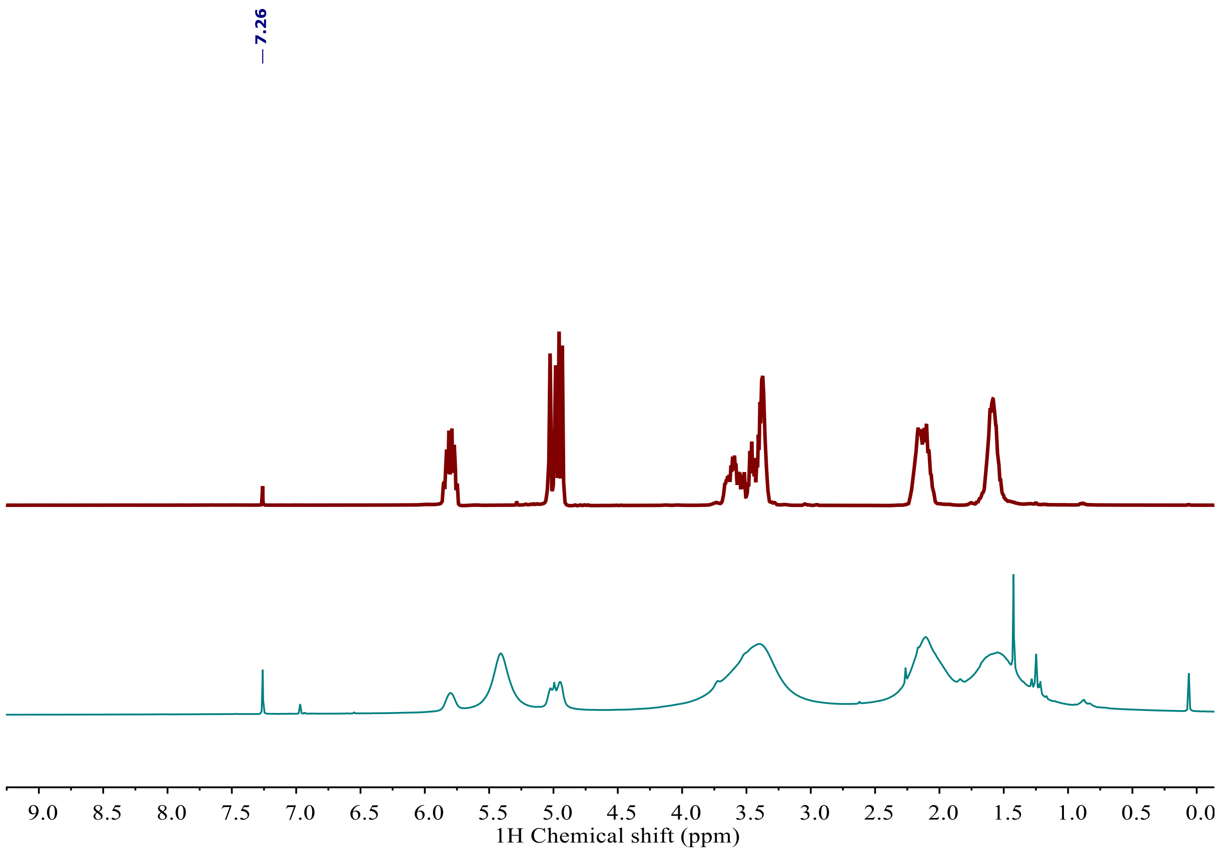

Figure S9: ^{1}H-NMR (*CDCl*$_3$, 500.2 MHz) of atactic PEH (top) and the metathesis product of PEH (88% con.) (bottom).

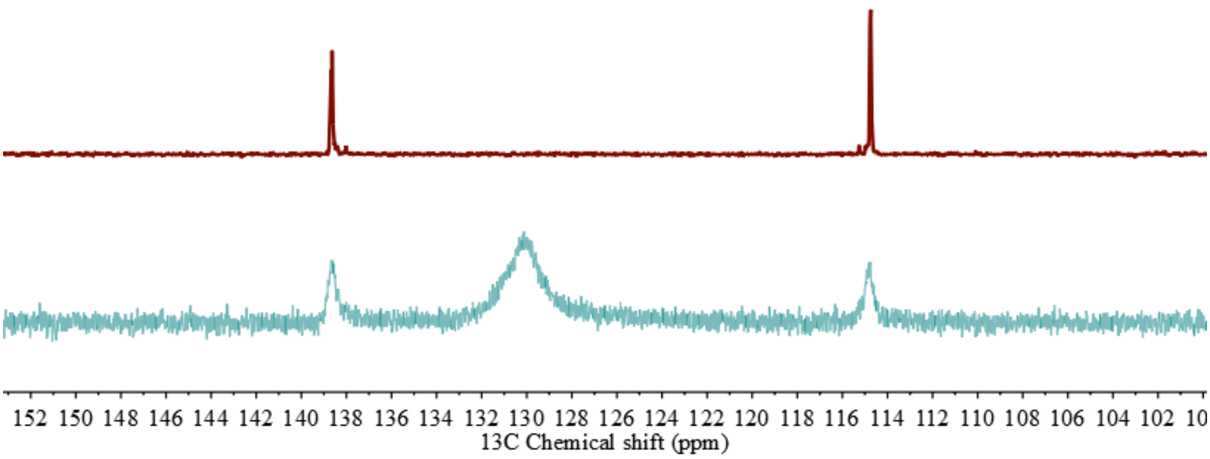

Figure S10: ^{13}C-NMR (*CDCl$_3$*, 125.8 MHz) of the olefin region of atactic PEH (top) and the metathesis product of PEH (88% con.)(bottom).

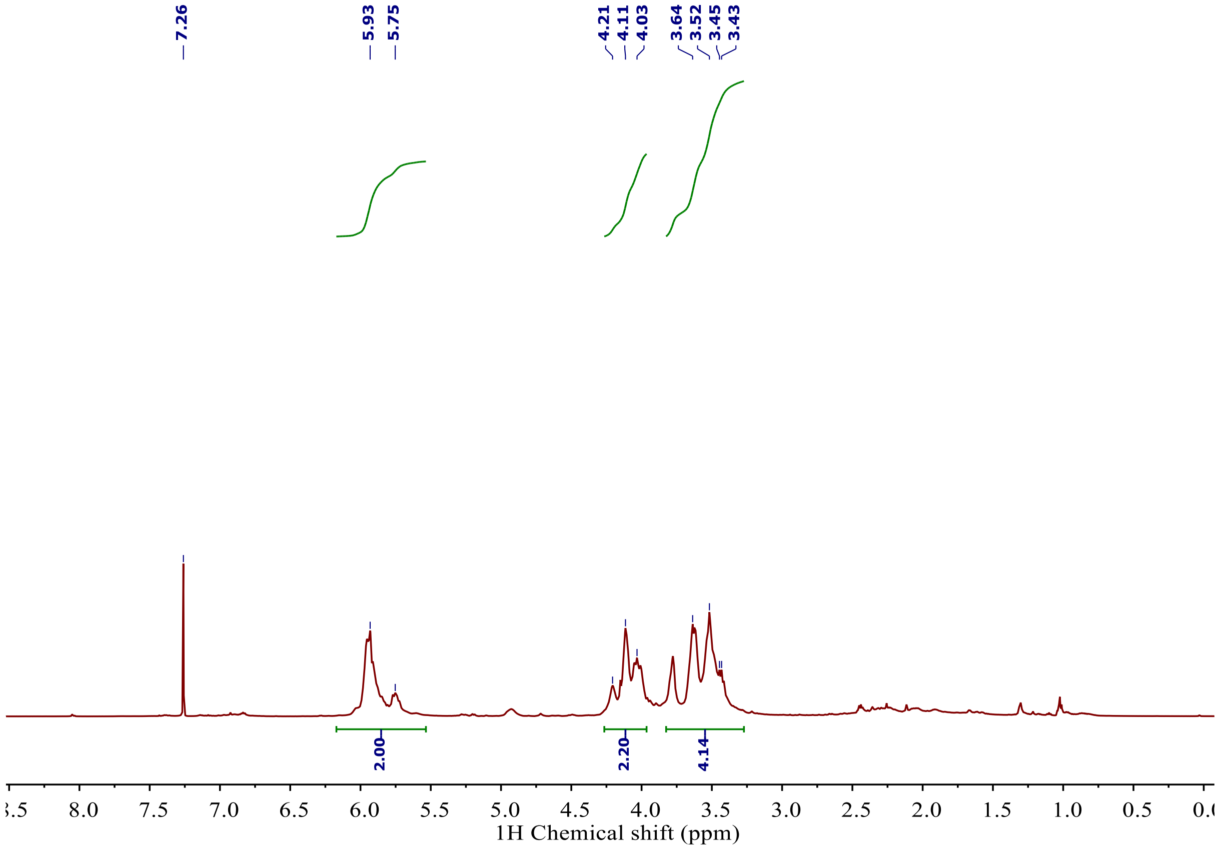

Figure S11: ^{1}H-NMR (*CDCl$_3$*, 500.2 MHz) of atactic functionalizable cyclopolyether.

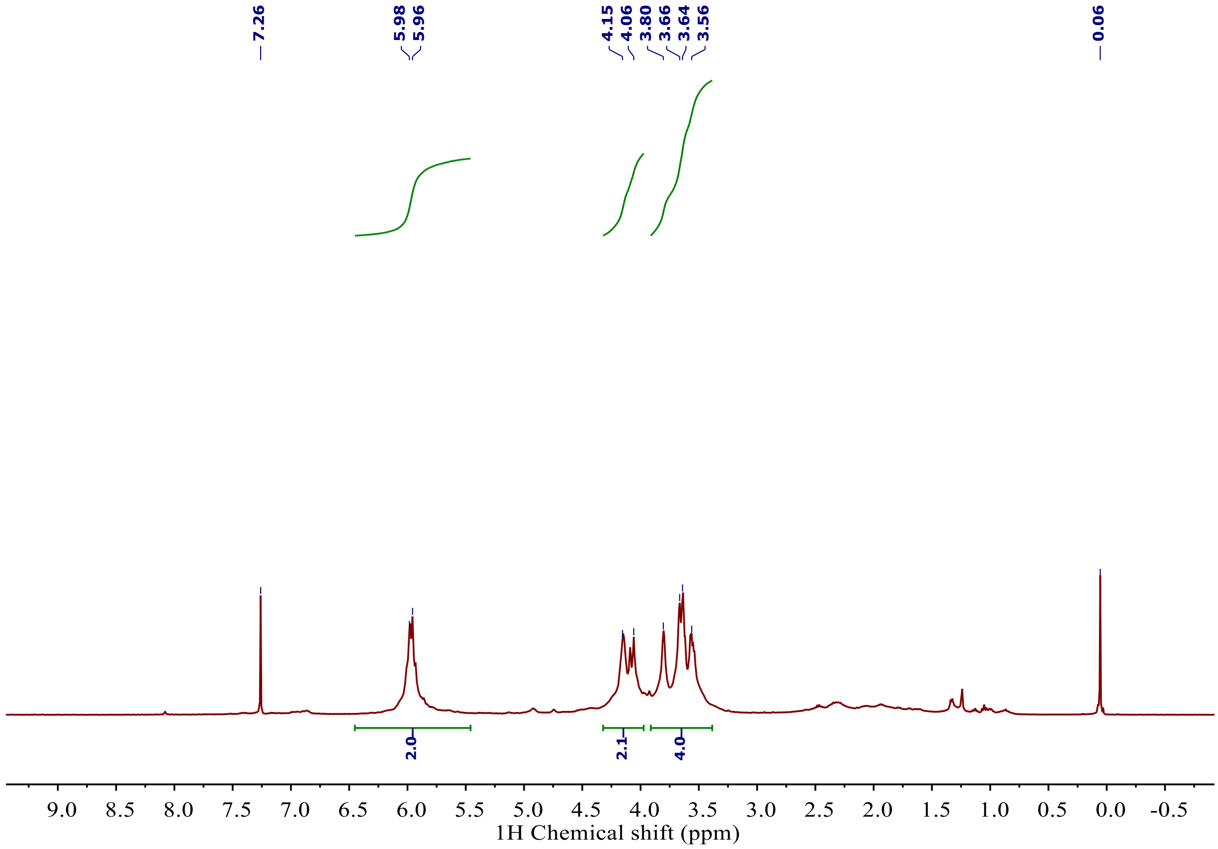

Figure S12: ^{1}H-NMR (*$CDCl_3$*, 500.2 MHz) of isotactic functionalizable cyclopolyether.

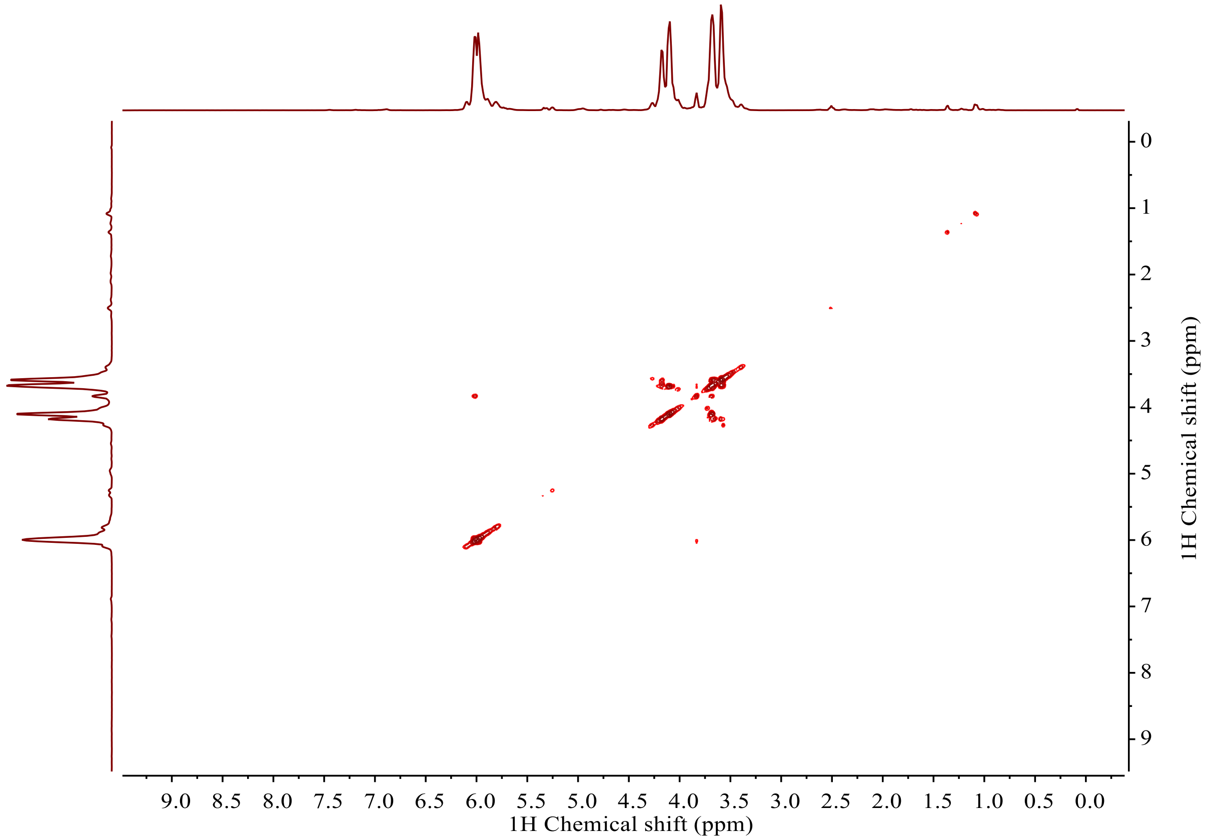

Figure S13: ^{1}H-^{1}H COSY NMR (*$CDCl_3$*, 500.2 MHz) of isotactic functionalizable cyclopolyether.

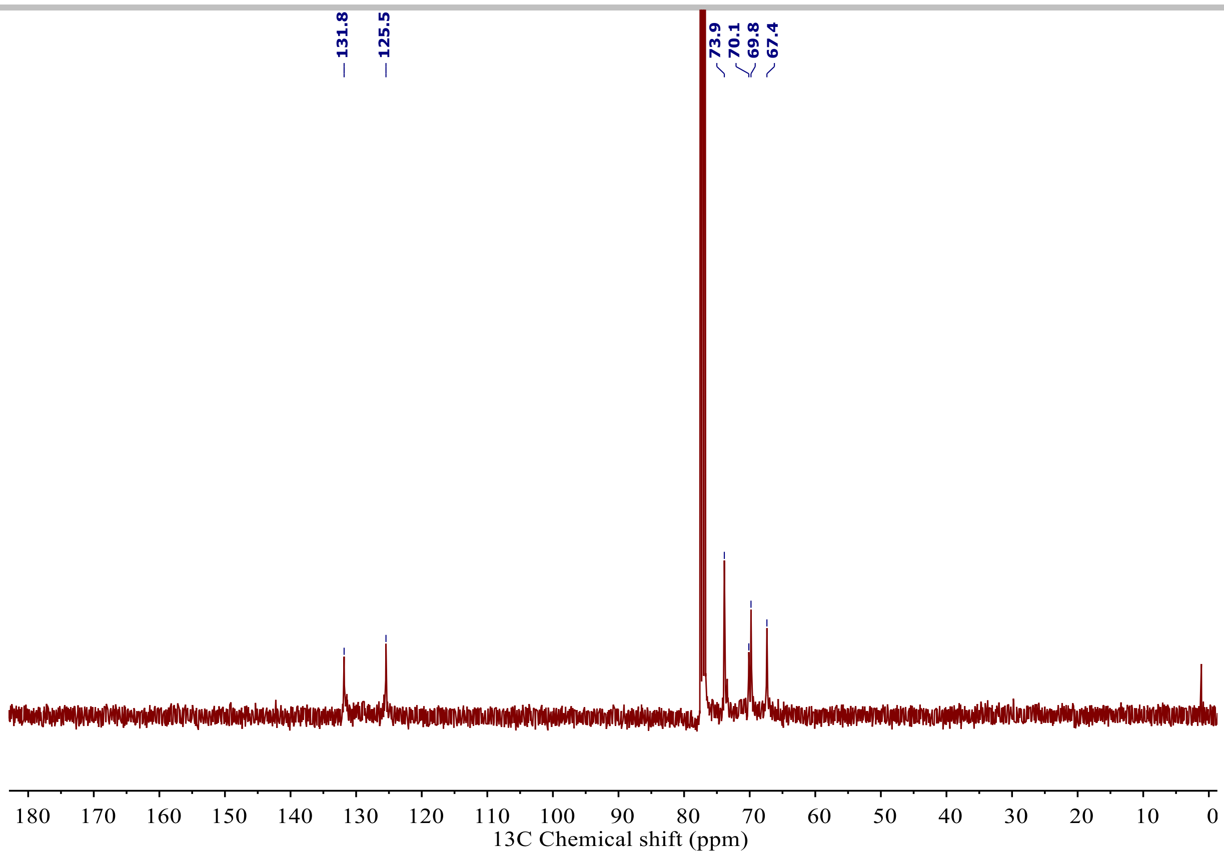

Figure S14: ^{13}C-NMR (*CDCl$_3$*, 125.8 MHz) of isotactic functionalizable cyclopolyether.

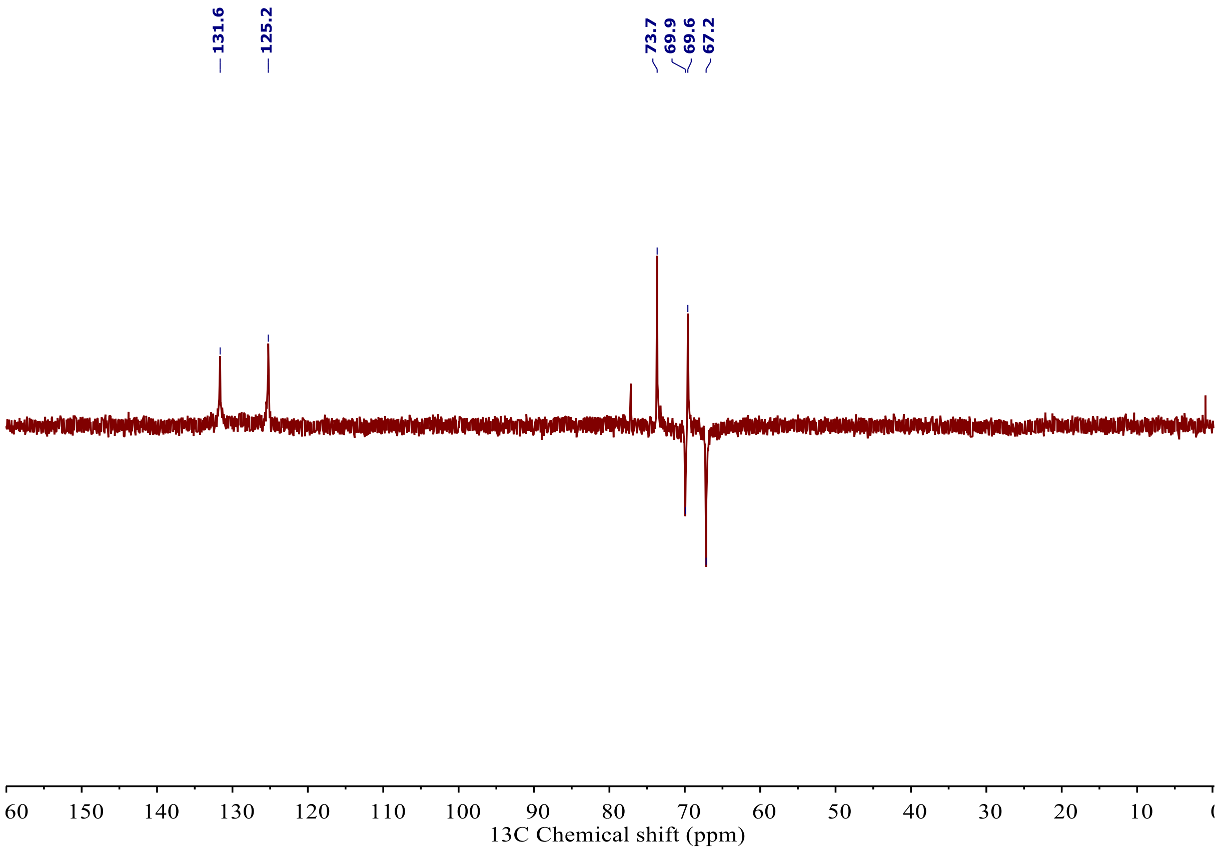

Figure S15: DEPT 135-NMR (*CDCl$_3$*, 500.2 MHz) of isotactic functionalizable cyclopolyether.

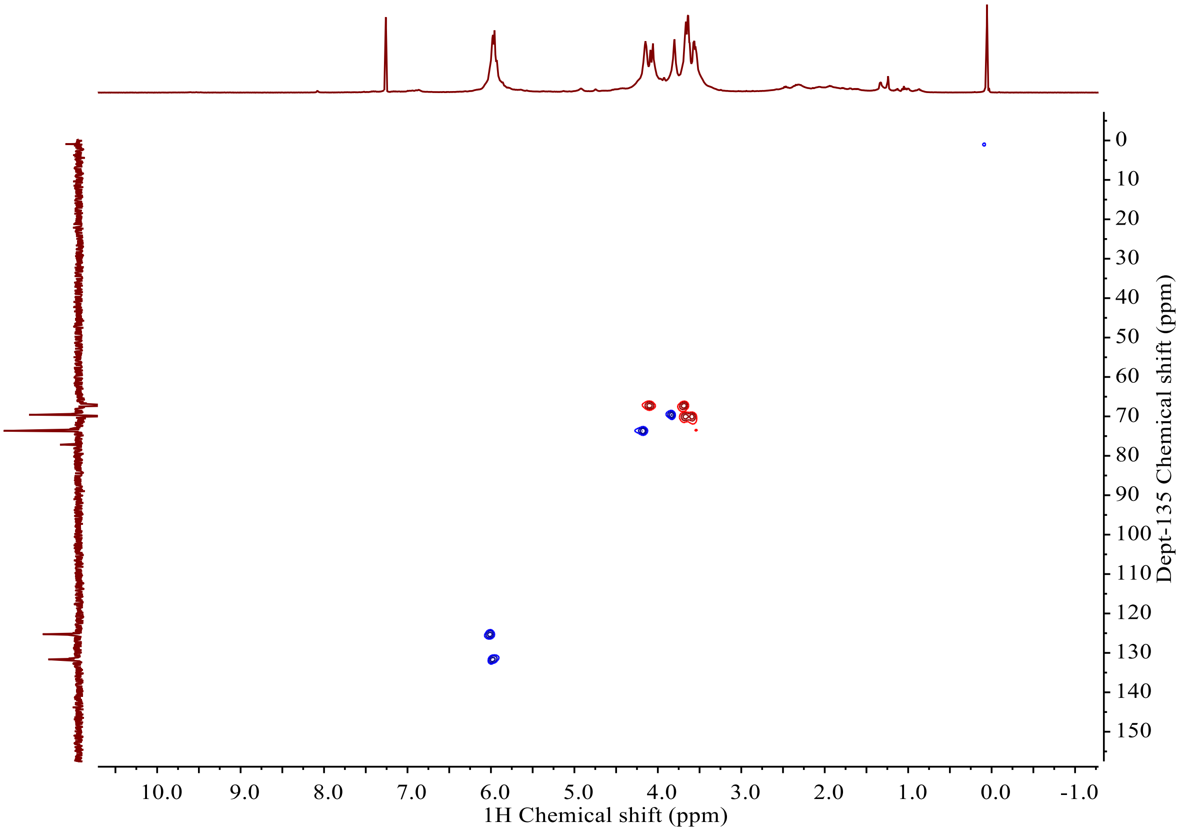

Figure S16: ^{1}H-Dept 135 HSQC (*$CDCl_3$*, 500.2 MHz) of isotactic functionalizable cyclopolyether.

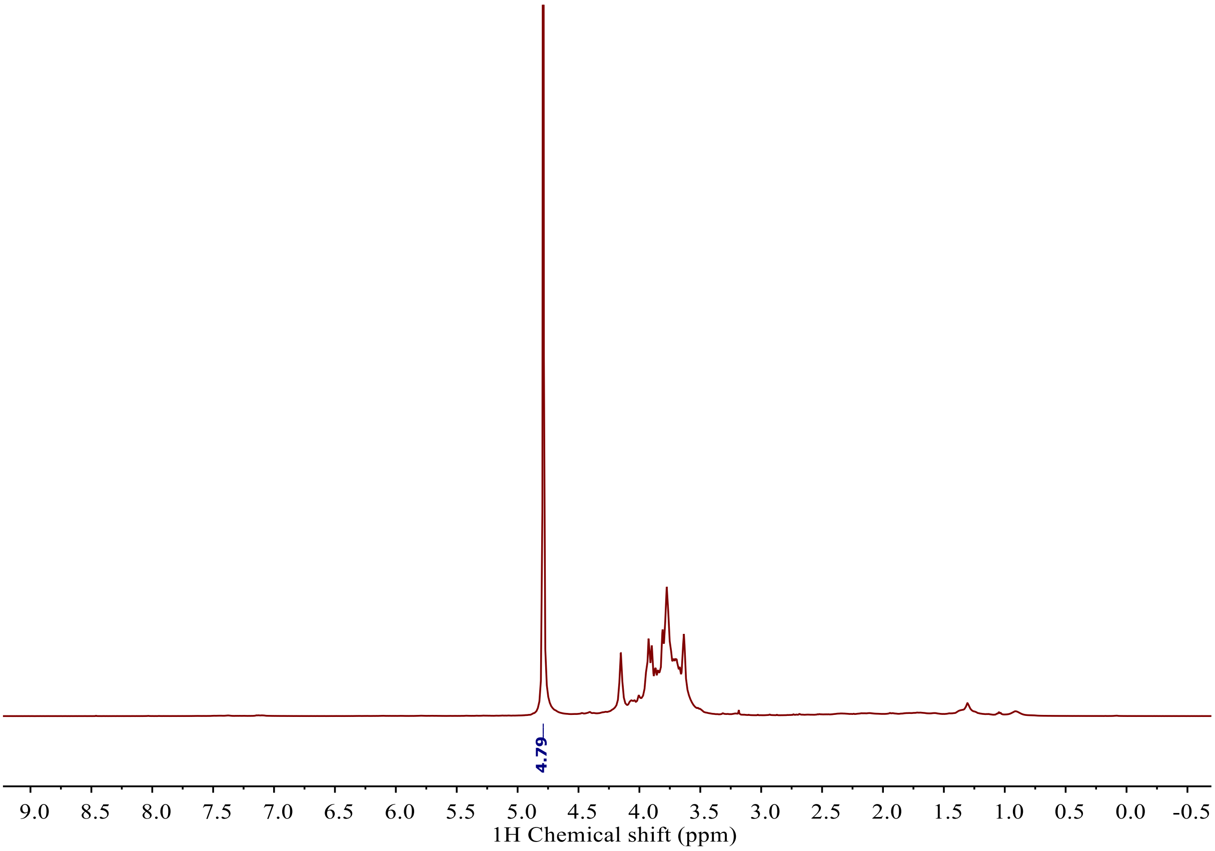

Figure S17: ^{1}H-NMR (*D_2O*, 500.2 MHz) of *R,R cis* PEGose.

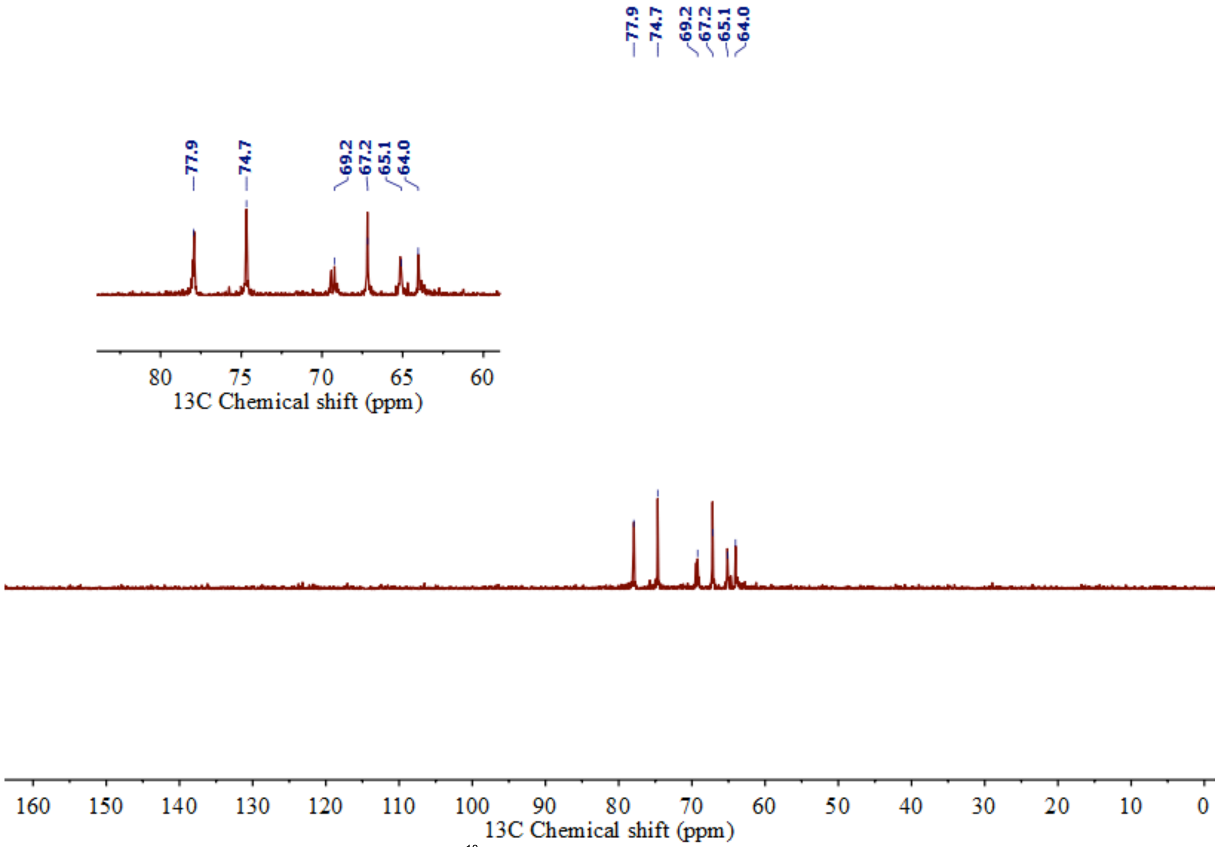

Figure S18: ^{13}C-NMR (*D_2O*, 125.8 MHz) of *R,R cis* PEGose.

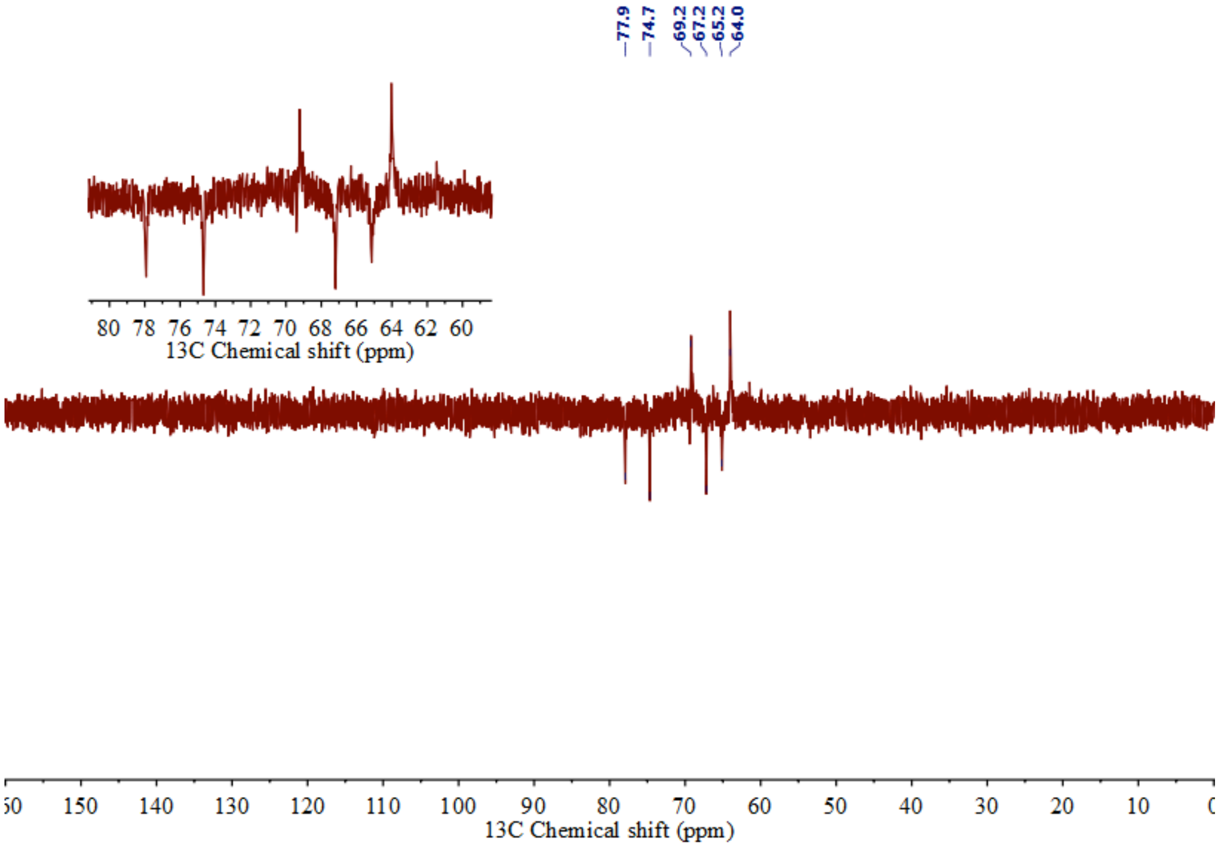

Figure S19: DEPT 135-NMR (*D_2O*, 125.8 MHz) of *R,R cis* PEGose.

FT-IR Spectroscopy

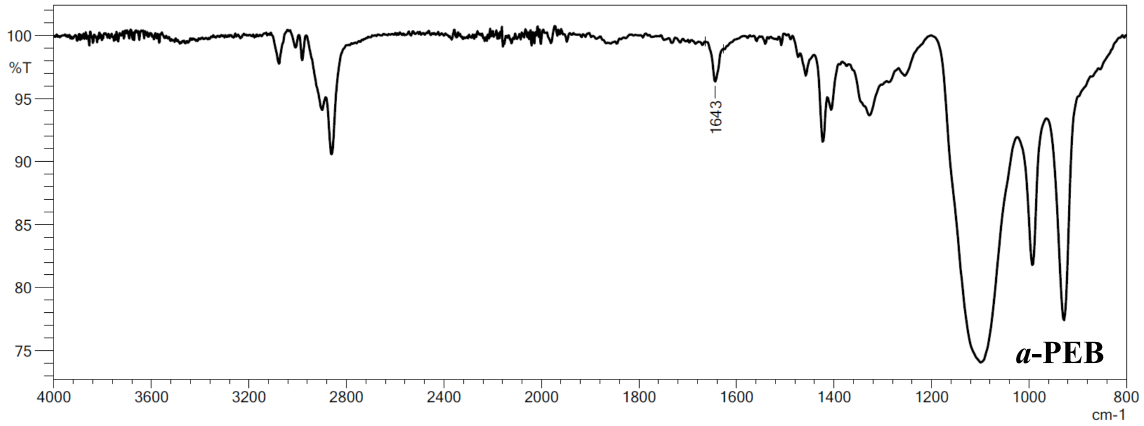

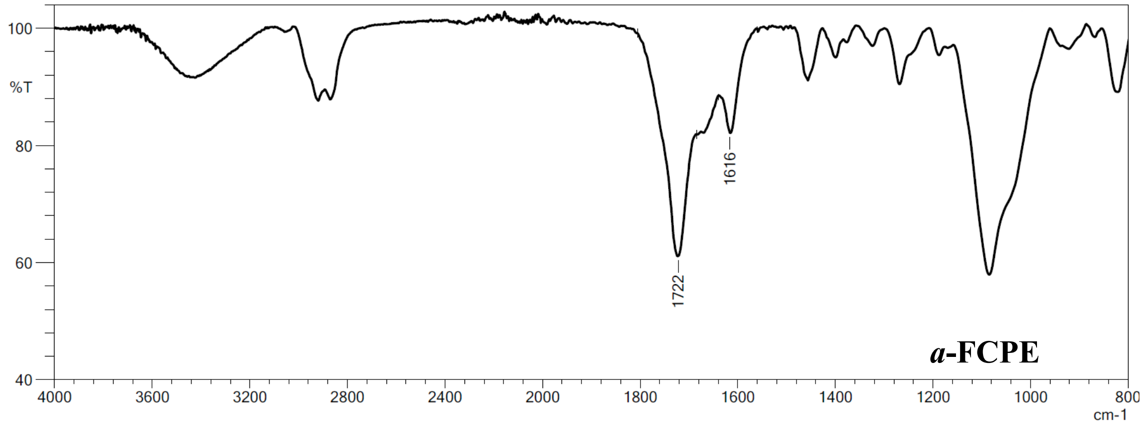

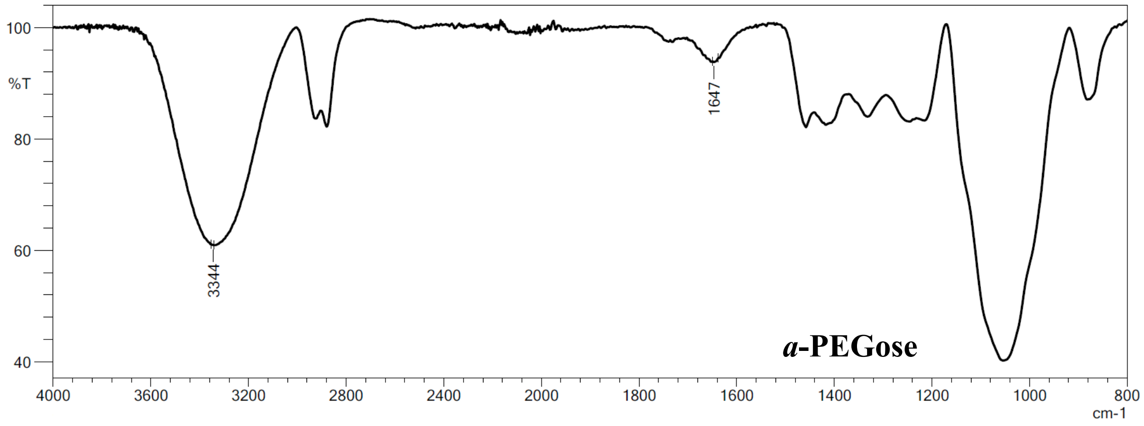

Differential scanning calorimetry

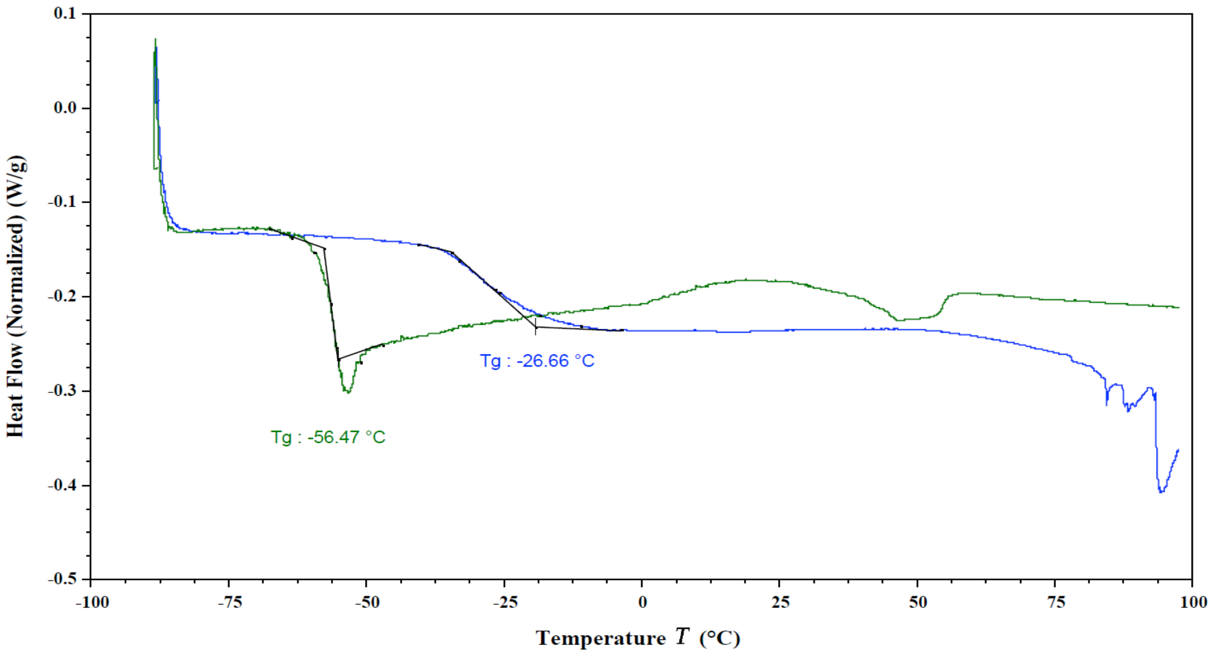

Figure S23: DSC thermogram of *a*-PEB (green) and *a*-FCPE (blue)

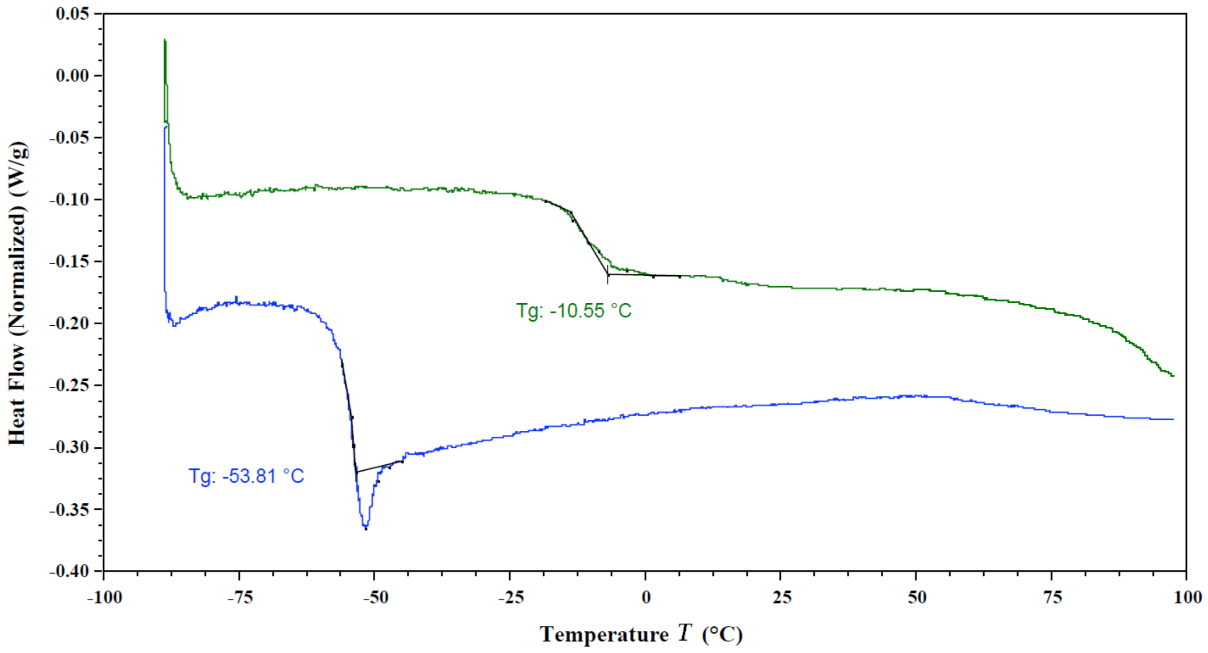

Figure S24: DSC thermogram of *i*-PEB (blue) and *i*-FCPE (green)

Inductively coupled plasma-optical emission spectrometry (ICP-OES) analysis:

The metals residues of Ru and Os in PEGose were measured by ICP-MS (Table S8). While the level of Ru was reduced significantly by 93.5% after using Sephadex column, Os traces decreased by 31% only. For this reason, PEGose was further purified to reduce the Os residues level by extraction using trioctylamine (TOA) as a scavenger. PEGose (100 mg) was dissolved in HCl 1M (2 mL) and TOA (2 mL) was added. The mixture was shacked strongly, and the pH was monitored to be acidic. The organic layer was then extracted by toluene (5 mL). This purification process was repeated twice and then the aqueous phase was naturalized by Na_2CO_3 and purified by the Sephadex G-25 column. Our novel scavenging method was extremely efficient using a very cheap scavenger TOA.

Table S8: The levels of Os and Ru metals per 100 mg of *R,R cis* PEGose.

Metal	Initial (mg)	After column (mg)	Purification efficiency (%)	After extraction (mg)	Purification efficiency (%)
**Os**	0.67	0.46 ± 0.01	31.4	0.0215 ± 10^{-3}	95.3
**Ru**	7.26	0.48 ± 0.01	93.5	-	-

Samples were analysed by ICP-MS using an Agilent 7500ce (with Octopole reaction system), employing an RF forward power of 1540 W and reflected power of 1 W, with argon gas flows of 0.81 L min^{-1} and 0.19 L min^{-1} for carrier and makeup flows, respectively. Sample solutions were taken up into the Micro mist nebuliser by peristaltic pump at a rate of approximately 1.2 mL min^{-1}. Skimmer and sample cones were made of nickel. The instrument was operated in spectrum acquisition mode and three replicate runs per sample were employed. The masses analysed were ^{188}Os and ^{189}Os for Os and ^{98}Ru for Ru. Each mass was analysed in fully quant mode (three points per unit mass). A series of standards was prepared using single element 1000 mg.L^{-1} (Qmx) diluted with 2% /0.5 % v/v HNO_3/HCl to give a range of standards.

Parameters for 'No gas' mode:
Ion Lenses:, Extract 1: 0 V, Extract 2: -133 V, Omega Bias-ce: -20 V, Omega Lens-ce: 0 V, Cell Entrance: -30 V, QP focus: 3 V, Cell Exit: -34 V.
Quadrupole Parameters:
OctP Bias: -6 V, QP Bias: -3 V

Circular dichroism (CD) spectra:

The circular dichroism (CD) spectra were measured at room temperature using a Jasco J-810 spectropolarimeter and a 0.02 cm path length quartz cuvette with *R,R* PEGose [2 $mg.mL^{-1}$] in deionised water at measurement range 260 - 180 nm. The ultraviolet photomultiplier parameters were set as follows; data pitch 0.2 nm, slit width 1 nm, response 2 s scan rate. Two scans for each sample were collected at a rate of 10 nm/min.

The reference was DI-water and traces of the catalysts used in PEGose preparation.

High absorbance and HT values precluded measurement below 180nm.

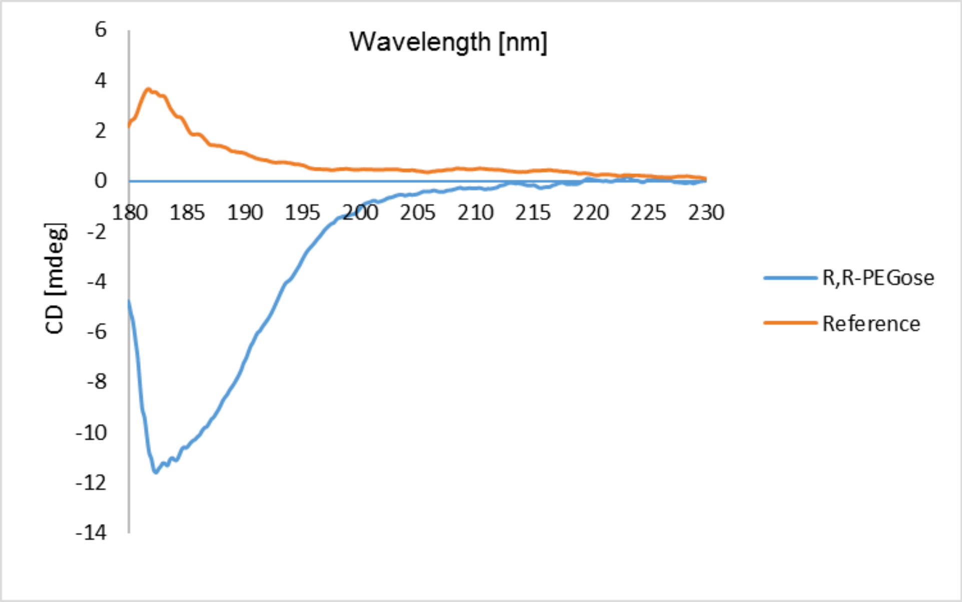

Figure S22: CD spectra of *R,R*-PEGose solution (blue) and reference (orange)

References

[1] Yasuda, T.; Aida, T.; Inoue, S. *Macromolecules,* **1982**, *16*, 1792–1796.

[2] Schaus, S. E.; Brandes, B. D.; Larrow, J. F.; Tokunaga, M.; Hansen, K.B.; Gould, A. E.; Furrow, M. E.; Jacobsen. E. N. *J. Am. Chem. Soc.,* **2002**, *124*, 1307–1315.

[3] Sugimoto, H.; Kuroki, M.;Watanabe, T. *Macromolecules*, **1993**, *1*, 3403–3410.

[4] Brocas, A. L.; Mantzaridis, C.; Tunc, D. & Carlotti, S. *Prog. Polym. Sci.,* **2013**, *38*, 845–873.

[5] Monfette, S.; Fogg, D. E. *Chem. Rev,* **2009**, *109*, 3783–3816.
